# Dissecting the heterogeneity of the alternative polyadenylation profiles in triple-negative breast cancers

**DOI:** 10.7150/thno.40944

**Published:** 2020-08-21

**Authors:** Lei Wang, Guan-Tian Lang, Meng-Zhu Xue, Liu Yang, Li Chen, Ling Yao, Xiao-Guang Li, Peng Wang, Xin Hu, Zhi-Ming Shao

**Affiliations:** 1 Department of Breast Surgery, Fudan University Shanghai Cancer Center, Fudan University, Shanghai 200032, People's Republic of China.; 2 Key Laboratory of Breast Cancer in Shanghai, Fudan University Shanghai Cancer Center, Fudan University, Shanghai 200032, People's Republic of China.; 3 Department of Oncology, Shanghai Medical College, Fudan University, Shanghai 200032, People's Republic of China.; 4 SARI Center for Stem Cell and Nanomedicine, Shanghai Advanced Research Institute, Chinese Academy of Sciences, Shanghai 201210, People's Republic of China.; 5 Bio-Med Big Data Center, CAS Key Laboratory of Computational Biology, CAS-MPG Partner Institute for Computational Biology, Shanghai Institute of Nutrition and Health, University of Chinese Academy of Sciences, Chinese Academy of Sciences, Shanghai 200031, People's Republic of China.; 6 School of Life Science and Technology, ShanghaiTech University, Shanghai 201210, People's Republic of China.; 7 Institutes of Biomedical Sciences, Fudan University, Shanghai 200032, People's Republic of China.

**Keywords:** alternative polyadenylation, subtype, triple-negative breast cancer, CPSF1, PABPN1

## Abstract

**Background:** Triple-negative breast cancer (TNBC) is an aggressive malignancy with high heterogeneity. However, the alternative polyadenylation (APA) profiles of TNBC remain unknown. Here, we aimed to define the characteristics of the APA events at post-transcription level among TNBCs.

**Methods:** Using transcriptome microarray data, we analyzed APA profiles of 165 TNBC samples and 33 paired normal tissues. A pooled short hairpin RNA screen targeting 23 core cleavage and polyadenylation (C/P) genes was used to identify key C/P factors.

**Results:** We established an unconventional APA subtyping system composed of four stable subtypes: 1) luminal androgen receptor (LAR), 2) mesenchymal-like immune-activated (MLIA), 3) basal-like (BL), 4) suppressed (S) subtypes. Patients in the S subtype had the worst disease-free survival comparing to other patients (log-rank *p* = 0.021). Enriched clinically actionable pathways and putative therapeutic APA events were analyzed among each APA subtype. Furthermore, CPSF1 and PABPN1 were identified as the master C/P factors in regulating APA events and TNBC proliferation. The depletion of CPSF1 or PABPN1 weakened cell proliferation, enhanced apoptosis, resulted in cell cycle redistribution and a reversion of APA events of genes associated with tumorigenesis, proliferation, metastasis and chemosensitivity in breast cancer.

**Conclusions:** Our findings advance the understanding of tumor heterogeneity regulation in APA and yield new insights into therapeutic target identification in TNBC.

## Introduction

Triple-negative breast cancer (TNBC), lacking estrogen receptor (ER), progesterone receptor (PR), and human epidermal growth factor receptor 2 (HER2) amplification, represents approximately 15-20% of primary breast cancers 1. TNBC is a highly heterogeneous disease with a high proliferative activity 2. TNBC patients' clinical courses are usually aggressive and the patients experience high rates of both early relapse and distant metastasis. Thus far, we have no specific and well-defined target therapies for TNBC, and surgery and chemotherapy remain the only therapeutic option. In recent years, identifying new potential therapeutic targets has become one of the hotspots in treating TNBC 3.

Alternative polyadenylation (APA) is a highly prevalent RNA-processing mechanism that generates distinct 3' ends on mRNAs and other RNA polymerase II transcripts 4. Recent studies have demonstrated that more than half of mammalian genes show APA which is an emerging layer of gene regulation 5-7. Tandem 3' untranslated region (3'UTR) APA, the most frequent APA form, results in transcripts with varying 3'UTR lengths without affecting the protein encoded by the gene 8, 9. APA events in the 3'UTR of mRNAs are correlated with multiple biological processes, including proliferation, tumorigenesis and differentiation 4, 10. Enhanced proliferation was associated with 3'UTR shortening and up-regulation of polyadenylation factors 11-13. However, the tandem 3'UTR APA events of TNBC has not been well characterized. Given the important role of APA events in proliferation and tumorigenesis, elucidation of APA patterns might improve our understanding of the nature of TNBC.

Even though TNBC is considered a single clinical entity, molecular profiling with 'omics' technologies have revealed an remarkably high level of heterogeneity as well as common features in TNBC 14. As an approach to decode the molecular patterns of cancers with complex genotypic characteristics, gene expression profiles have been analyzed. A more comprehensive understanding of cancer etiology can be obtained by identifying a series of “driver” signaling pathways (for example, cell cycle, DNA damage response, and immune cell processes). Due to high-throughput technologies, emerging roles of the transcriptome have been identified in the cellular processes that are associated with carcinogenesis. Different groups independently reporting genomic profiling of TNBC identified four to six intrinsic subtypes displaying unique gene signatures and ontologies 15-17. Thus, we hypothesize that TNBC has the interpatient heterogeneity in APA profiles.

However, whether APA patterns can be utilized in TNBC subtyping is unclear. In this study, we analyzed the transcriptome array data of 165 TNBC and 33 normal adjacent tissues for patterns of APA and gene expression and identified 4 stable TNBC subtypes: (i) luminal androgen receptor (LAR), (ii) mesenchymal-like immune-activated (MLIA), (iii) basal-like (BL), and (iv) suppressed (S). Furthermore, using pooled short hairpin RNA (shRNA) library screening, we identified CPSF1 and PABPN1 as the key regulators of cell proliferation and APA in TNBC. These results reveal an active role of the 3'UTR in regulating biological processes and provide a potential therapeutic target for TNBC.

## Methods

### Patients and specimens

This prospective observational study was initiated on January 1, 2011. A total of 165 consecutive patients, treated in the Department of Breast Surgery at Fudan University Shanghai Cancer Center (FUSCC, Shanghai, China) from January 1, 2011 to December 31, 2012, were recruited according to the following inclusion criteria: (i) female patients diagnosed with unilateral disease; (ii) patients with histologically confirmed invasive ductal carcinoma (IDC) with a triple-negative (ER^-^/PR^-^/HER2^-^) phenotype; (iii) patients with no evidence of metastasis at diagnosis; and (iv) patients with no treatment prior to surgery. The exclusion criteria were as follows: (i) ductal carcinoma *in situ* (with or without microinvasion); (ii) inflammatory breast cancer; and (iii) percentage of tumor cells less than 80%. The ER, PR, and HER2 status was assessed individually by two pathologists at the Department of Pathology in FUSCC according to the American Society for Clinical Oncology/College of American Pathologists guidelines 18, 19. We also collected 33 paired adjacent normal breast tissues from the FUSCC tissue bank. Tissue samples were obtained with the approval of an independent ethical committee / institutional review board at FUSCC, Shanghai Cancer Center Ethical Committee (Shanghai, China), and written informed consent was provided by all patients.

### Microarray data

Total RNA was isolated from 165 frozen TNBC samples and 33 adjacent normal tissues using the RNeasy Plus Mini Kit (Qiagen). The Affymetrix GeneChip Human Transcriptome Array 2.0 (HTA 2.0) was used to quantify transcriptome expression profiles after quality control according to the manuals. The CEL files were processed with 'aroma.affymetrix' using RMA background correction and quantile normalization 20. The processed probe intensities were extracted from the intermediate CEL files and log2 transformed to allow probe-level analysis. A PLATA-like approach was used to normalize the probe-level data as previously described 12, 21. The probe intensities were normalized to the median intensity of all probes mapped to the transcript in each sample. Gene expression analyses are detailed in **Supplementary Methods**. Microarray data were deposited into the Gene Expression Omnibus (GEO) database (GSE76250).

### Tandem 3′UTR analysis

To identify the set of transcripts with tandem 3'UTRs that could be profiled with the Affymetrix GeneChip HTA 2.0, we first queried the Ensembl database with the following filters: 1) transcript count ≥ 4; and 2) HTA 2.0 probeset IDs. We then retrieved the 3'UTRs of the returned Ensembl Transcript IDs using Ensembl annotation data (Ensembl release 75, GRCh37.p13). The genomic coordinates of each 3'UTR were compared. Transcripts with the same 3'UTR start position and different APA sites were identified as tandem 3'UTRs. The probes of HTA 2.0 were mapped to the hg19 genome and the uniquely mapped probes in each tandem 3′UTR were kept. The tandem 3'UTRs with at least four probes before and after APAs were retained for subsequent 3'UTR APA analysis, resulting in 15,264 tandem 3'UTRs. The sample probe intensities were first normalized to the median probe intensities of the normal sample. The probes were partitioned into common and extended groups using Bayesian analysis of the change point (BCP), which is implemented by R package 'bcp' (version 4.0.0)^22^. This approach treated all samples with a tandem 3'UTR as a multivariate series with a common change point. The algorithm input is an *n* (number of probes for the 3'UTR) by *m* (number of samples) matrix. The probe position with the largest posterior probability was identified as the change point. The short 3′UTR index (SUI) for sample *i* was defined as following:


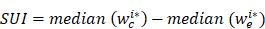


where wi* *c* and wi* *e* are the posterior probabilities of common and extended regions for sample *i*. The larger the value of the SUI, the higher the proportion of the short 3′UTR isoform is and *vice versa*. Because 89% of the significant changes in isoform abundance occur at the first functional APA site 23, we focus our analyses on genes with one APA site. We discarded the tandems with more than one peak of posterior probability (multiple APAs) and manually examined Bayesian change point plots. After manual verification, the following tandem 3'UTRs were excluded: (1) tandem 3'UTRs with multiple peaks of posterior probability (multiple APAs); (2) tandem 3'UTRs with instable posterior means before or after the change point. As a result, 2,869 tandem 3′UTRs were obtained for subsequent analyses.

### The tandem-3′UTR-based TNBC subtyping

Tandem 3′UTRs were sorted by the coefficient of variation (CV) of SUI across all samples and the top 25% most variant tandem 3′UTRs were chosen for the subsequent clustering analysis. The unsupervised non-negative matrix factorization (NMF) method was used to determine the optimal number of stable TNBC subtypes. We used a non-smooth NMF (nsNMF) algorithm and applied 50 and 200 iterations for the rank survey and the clustering runs, respectively. The ideal rank basis and factorization was determined based on the review of consensus matrices, the cophenetic and dispersion coefficients and silhouette widths from clustering solutions with 2 to 10 ranks. The robustness of the classification system was verified by consensus clustering, which involves k-means clustering by resampling (1,000 iterations) randomly selected tumor profiles. Visual representation of the consensus matrix reveals the proportion of times in which two samples are clustered together across the resampling iterations. To determine the optimal number of tandem 3'UTRs in NMF clustering, we examined the clustering results by selecting genes based on the absolute CV from the top 5% to the top 45% and finally chose the top 25% absolute CV to perform NMF clustering (**Figure S1**).

The R package 'NMF' (version 0.20.6) was used to perform the NMF analysis. We then used MATLAB 2015a (MathWorks, Natick, MA, USA) to conduct the principal component analysis (PCA).

### Extrapolating the clustering results to TNBC cell lines

We extrapolated the APA-based subtypes TNBC cell lines, including MDA-MB-231 and MDA-MB-468, using the R package 'pamr' 24. Briefly, 'pamr' uses nearest shrunken centroids method to assign the subtypes of each cell line. The SUI values were normalized using quantile normalization before we using the 'pamr.train' and 'pamr.predict' functions.

### The Lehmann/Pietenpol classification

We assigned TNBC samples to Lehmann/Pietenpol subtypes using the TNBCtype tool (http://cbc.mc.vanderbilt.edu/tnbc/) 15, 25. Fisher's exact test was implemented to evaluate the relationship between the Lehmann/Pietenpol subtyping system and our tandem-3′UTR-based system.

### Reverse phase protein arrays (RPPA) data

The RPPA data (level 4) of breast cancer samples from the Cancer Genome Atlas (TCGA) were downloaded from the Cancer Proteome Atlas (TCPA, https://tcpaportal.org/tcpa/) 26, 27.

### Pathway and co-expression network analyses

See **Supplementary Methods.**

### Pooled shRNA screening

pLKO.1 lentiviral plasmids encoding shRNAs targeting 3' processing factors and nontargeting controls were each used as a pool. shRNA lentiviruses were designed according to the information in the RNAi Consortium (Broad Institute of MIT and Harvard) and generated from HEK293T cells. As previously reported 28, MDA-MB-231 and MDA-MB-468 cells were infected with lentiviral supernatant containing shRNAs with a multiplicity of infection (MOI) of 0.3. Detailed methods are provided in **Supplementary Methods.**

### Cell culture, stable cell line construction and Western blotting

See **Supplementary Methods.**

### Cell proliferation, apoptosis and cell cycle analyses

See **Supplementary Methods.**

### RNA-seq data analysis and profiling APA events from RNA-seq data

See **Supplementary Methods.**

### Statistical analysis

All experiments were repeated at least three times. The data are presented as the mean of biological replicates unless otherwise indicated. Error bars represent the standard deviation from the mean, unless otherwise indicated. Continuous variables were analyzed using *t* test, Mann-Whitney test or analysis of variance (ANOVA). The time-dependent receiver operating characteristic (ROC) curve analysis was performed using R package 'timeROC' 29. All statistical analyses were completed using R 3.2.3 (R Development Core Team, Vienna, Austria) with two-sided tests. *P*-value < 0.05 was considered to be statistically significant. Detailed methods are provided in **Supplementary Methods.**

## Results

### Characterization of the tandem 3'UTR landscape in TNBC

**Figure 1:**
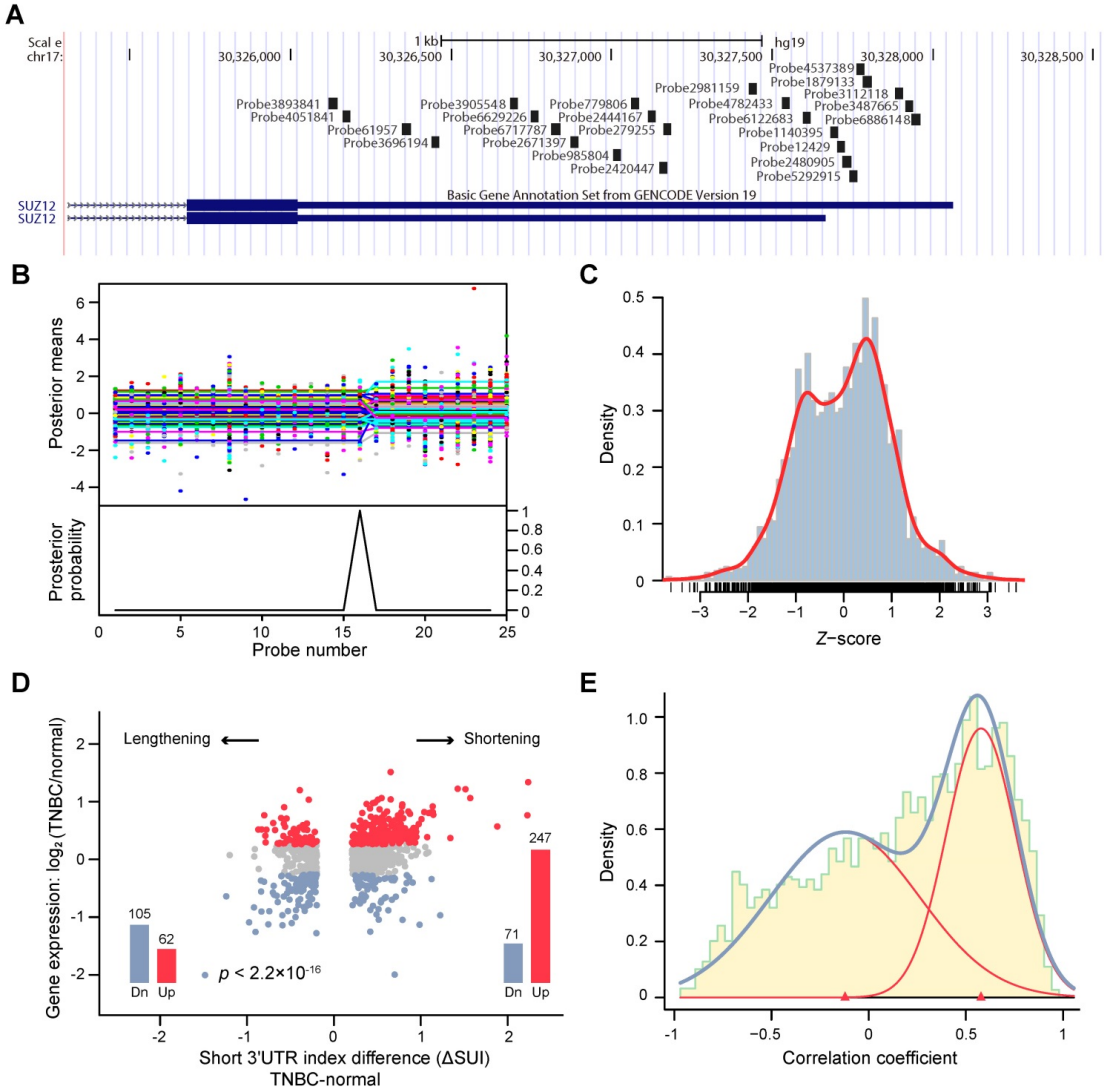
Bayesian change point (BCP) approach for alternative polyadenylation (APA) analysis of transcriptome array data. (**A**) Mapping of Human Transcriptome Array 2.0 probes to the *SUZ12* 3′UTR. (**B**) BCP analysis results. The upper panel represents the input probe intensities (dots) and posterior probe mean intensities (lines) for all samples. The lower panel reveals the probabilities of the posterior change point. (**C**) The histogram represents the distribution of the *z*-score of the short 3′UTR index (SUI) and the red line is the estimated density. (**D**) The fold-changes in expression between TNBC and normal adjacent tumor tissues are plotted against the ΔSUI values. The genes significantly up- and down-regulated in TNBC are shown in red and blue, respectively. The red and blue bars indicate the number of up- and down-regulated genes, respectively. (**E**) The correlation coefficient between 3′UTR shortening and gene expression follows a bimodal distribution. The histogram represents the distribution of the correlation coefficient and the estimated densities are shown for individual (red line) and combined (blue line) distributions. Abbreviations: 3′UTR, 3′ untranslated region; APA, alternative polyadenylation; BCP, Bayesian change point; HTA, human transcriptome array; SUI, short 3′UTR index.

We aimed to profile the genome-wide APA patterns in the TNBC transcriptome. To achieve this, we developed algorithms based on a Bayesian approach to analyze tandem 3'UTR APA events. We focused on tandem 3'UTRs because a series of studies have identified their active roles in carcinogenesis 13, 30, 31. Using transcriptome arrays of 165 tumors and 33 paired normal adjacent tissues, we characterized the tandem 3'UTR landscape of TNBC. The transcriptome array probes were first mapped to hg19 using PLATA-like methods 12, 21 (**Figure 1A**). The tandem 3'UTRs were profiled using a multivariate Bayesian approach, which considered all samples in the same calculation (see **Methods**). This algorithm assessed the posterior probability and mean of each probe as the change point in the same Bayesian procedure, which substantially reduced the number of statistical calculations (**Figure 1B**;**Figure S2**). We termed the estimated 3'UTR shortening or lengthening as the “short 3'UTR index” (SUI). Because 89% of the significant changes in APA isoform expression occur at the first APA site 23, we focused on genes with one APA site in this study. After computational selection and manual verification, 2,869 tandem 3'UTRs with only one significant APA site were identified (**Figure S3**). **Figure 1C** shows the distribution of the *z*-score of the SUI.

To investigate the consequence of tandem 3'UTR APA events, we compared gene expression between TNBC and adjacent normal tissues. As shown in **Figure 1D**, genes with shorter 3'UTRs in cancers are likely to be more highly expressed in cancer samples than those with longer 3'UTRs, which suggests that transcripts losing elements in the 3'UTR prone to be upregulated. Using a large dataset, we determined the correlation coefficient between tandem 3'UTR APA events (measured by the SUI) and gene expression, which apparently followed a bimodal distribution (**Figure 1E**). The majority of genes demonstrated a strong positive correlation, which supports the negative correlation between 3'UTR length and gene expression. Out of 1631 significant APA events, 68.5% (1118 of 1631) were 3′UTR shortening, whereas 31.5% (513 of 1631) were 3′UTR lengthening events (**Figure S4A**). Gene Ontology (GO) enrichment analysis of genes with APA events revealed enrichment in overall proliferation-associated functions and metabolic pathways (**Figure S4B-C**). This is consistent with the microarray study of APA events in breast cancer conducted by Akman et al 32. To compare the difference of APA quantification between microarray data (SUI) and RNA-seq data (percentage of distal poly(A) site usage index, PDUI), microarray assays coupled with RNA-seq were performed on MDA-MB-231. We observed a weak correlation between the two data sets (ρ = - 0.0575, *p* < 0.001; **Figure S5**). Based on the SUI and the PDUI calculation formula, the SUI depicted differential length of tandem 3'UTR in tumor tissues relative to normal tissues whereas the PDUI reflected the proportion of distal poly(A) site (PAS) of a transcript in a sample regardless of normal control. Furthermore, analysis of RPPA data and PDUI data from TCGA breast cancer cohort revealed a weak correlation between protein expression and APA events in 41 / 175 proteins (**Figure S6**; **Table S1**). The highest correlation coefficient was observed for PARP1 (*r* = -0.37, false discovery rate [FDR] < 0.001).

### Profiling tandem 3′UTRs of TNBC reveals four stable subtypes

**Figure 2:**
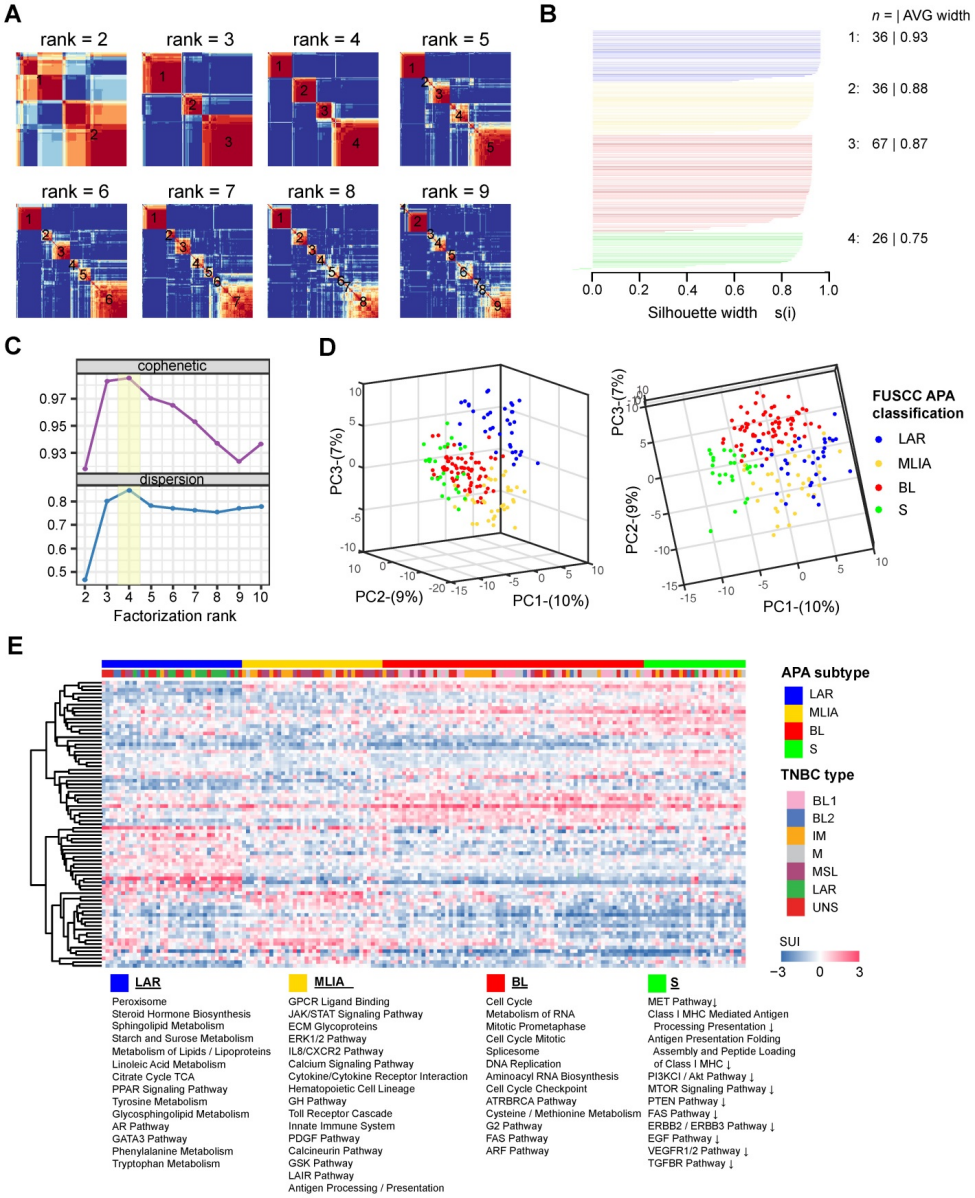
Classification of triple-negative breast cancers (TNBCs) by alternative polyadenylation (APA) profiling reveals 4 stable subtypes. (**A**) Consensus heatmaps showing the robustness of sample classification using non-negative matrix factorization (NMF) clustering. (**B**) Silhouette plot displaying the composition (*n* = number of samples) and stability (average width) of clustering. (**C**) Cophenetic and dispersion metrics for NMF across 2 to 10 clusters with 50 runs suggest 4 stable subtypes. (**D**) Principal component analysis depicts fundamental differences in the short 3′UTR index (SUI) between the TNBC APA subtypes. (**E**) The heatmap displaying the top 20 differentially polyadenylated 3′UTRs with most different SUIs in each subtype. Abbreviations: APA, alternative polyadenylation; NMF, non-negative matrix factorization; TNBC, triple-negative breast cancer; SUI, short 3′UTR index.

A total of 165 TNBC patients were recruited in this study based on inclusion and exclusion criteria. Using tandem 3′UTR profiling, we explored the TNBC molecular phenotypes. The NMF method was performed on 717 tandem 3′UTRs with the top 25% CV of the SUI across all samples to optimize the separation of stable intrinsic subtypes. After visual inspection of the consensus heatmaps (**Figure 2A**), the TNBC samples were most stably divided into 4 clusters by silhouette, cophenetic and dispersion metrics (**Figure 2B and C**). Unsupervised dimension reduction using PCA revealed fundamental differences in the tandem 3'UTR APA events between TNBC intrinsic subtypes identified by NMF and consensus clustering (**Figure 2D**). Tandem 3′UTRs with different SUIs from each subtype (Benjamini-Hochberg adjusted *p* < 0.001 from linear models for microarray and RNA-seq data) were computed and analyzed for pathway enrichment. **Figure 2E** shows the 3′UTR signature for these canonical pathways enriched in each TNBC subtype.

#### Subtype 1: the luminal androgen receptor (LAR) subtype

**Figure 3:**
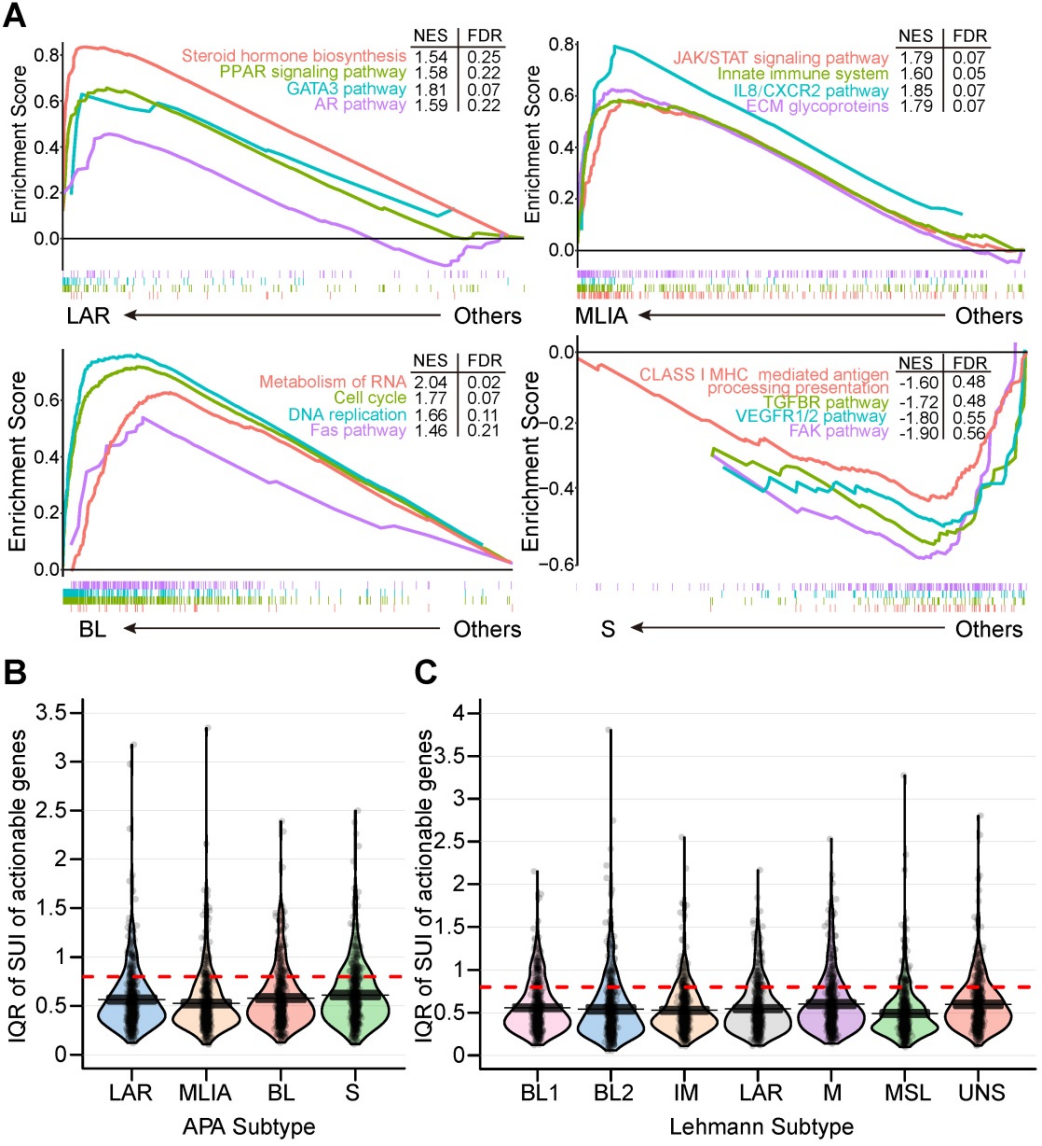
Alterations of alternative polyadenylation (APA) events in clinically actionable therapeutic targets in triple-negative breast cancer (TNBC). (**A**) Gene Set Enrichment Analysis (GSEA) of four APA subtypes (LAR, MLIA, BL and S). (**B**) Distribution of the interquartile range (IQR) of the short 3′UTR index (SUI) of clinically actionable genes across APA subtypes. (**C**) Distribution of the IQR of the SUI of clinically actionable genes across Lehmann subtypes. Abbreviations: APA, alternative polyadenylation; BL, basal-like; FDR, false discovery rate; GSEA, Gene Set Enrichment Analysis; IQR, interquartile range; LAR, luminal androgen receptor; MLIA, mesenchymal-like immune-activated; NES, normalized enrichment score; S, suppressed; SUI, short 3′UTR index; TNBC, triple-negative breast cancer.

Subtype 1 TNBCs displayed heavily enriched hormonally regulated pathways (**Figure 2E and 3A**) but ER-negative immunochemical staining. Steroid hormone biosynthesis, the AR pathway, oxidative phosphorylation and the peroxisome proliferator-activated receptor (PPAR) signaling pathway were significantly activated in this subtype. Notably, twenty-eight enriched pathways of subtype 1 were elevated in the LAR subtype of the Lehmann/Pietenpol classification as well. As shown in **Figure S7**, subtype 1 is uniquely enriched in AR-driven genes (*AR*, *DHCR24*, *ALCAM*, *FASN*, *FGFR4*, *FKBP5*, *SPDEF*, *SPOD*, *PIP* and *CLDN8*) and cytokeratin genes (*KRT8*, *KRT18* and *KRT19*). The results suggest that tumors in subtype 1 exhibit luminal gene expression patterns and may respond to anti-androgen and anti-estrogen therapies. In concordance with previous studies 15, we termed subtype 1 the LAR subtype.

#### Subtype 2: Mesenchymal-like immune-activated (MLIA) subtype

Subtype 2 exhibited a variety of signaling pathways combining mesenchymal-like and immunomodulatory gene expression patterns. Enriched pathways in the MLIA subtype included extracellular matrix (ECM) glycoproteins, the JAK/STAT signaling pathway, the ERK1/2 pathway, cytokine-cytokine receptor interactions, the platelet-derived growth factor (PDGF) pathway, the IL8/CXCR2 pathway, the innate immune system and antigen procession and presentation pathways (**Figure 2E and 3A**). As illustrated in **Figure S7**, cell growth and differentiation is characterized by elevated expression of growth factors (*FGF1*, *FGF2*, *FGFR1*, *FGFR2*, *IGF1*, *IGF2*, *IGFBP4*, *IGFBP5*, *IGFBP6*, *IGFBP7*, *NGFR*, *PDGFA*, *PDGFRA*, *PDGFRB*, *PDGFC*, *PDGFD* and *IGF1R*), epithelial-mesenchymal transition (EMT)-associated genes (*MMP2*, *ACTA2*, *SNAI2*, *SPARC*, *TAGLN*, *TWIST1*, *ZEB1*, *COL3A1*, *COL5A2*, *GNG11*, *ZEB2* and *CDH1*), Wnt signaling (*DKK2*, *SFRP4*, *TCF4*, *FZD4*, *CAV1*, *CAV2* and *CCND2*), and angiogenesis genes (*KDR*, *TFK*, *TIF1* and *EPAS1*). In addition, the MLIA subtype displayed low levels of genes related to cell proliferation, DNA damage response and cytokeratin, which were accompanied by enrichment of immune signal transduction genes (*IRF8*, *IRF1*, *IRF7*, *ITK*, *JAK1*, *JAK2*, *LCK*, *LYN*, *NFKB1*, *NFKBIA*, *STAT4*, *STAT5A*, *BTK* and *ZAP70*), stem-cell associated genes (*ABCA8*, *PROCR*, *ENG*, *PER1*, *ABCB1*, *TERF2IP*, *BCL2*, *BMP2*, *THY1*, *NT5E* and *VCAM1*) and HOX genes (*MEIS1*, *MEIS2*, *MEOX1*, *MEOX2* and *MSX1*). Thus, we named subtype 2 the MLIA subtype.

#### Subtype 3: Basal-like (BL) subtype

The top gene ontologies for subtype 3 were remarkably enriched in cell cycle and cell division related pathways (cell cycle, cell cycle checkpoints, G1 to S cell cycle, DNA replication, DNA repair), which represent distinct basal-like signatures (**Figure 2E and 3A**). Tumors of this subtype comprised the highest proportion of TNBCs (40.6%). Increased proliferation genes (*AURKA*, *AURKB*, *CENPA*, *CENPF*, *BUB1*, *TTK*, *CCNA2*, *PRC1*, *MYC*, *NRAS*, *PLK1*, *BIRC5* and *MKI67*) were observed, which was consistent with the high expression levels of DNA damage genes (*CHEK1*, *FANCA*, *FANCG*, *RAD54B*, *RAD51*, *NBN*, *EXO1*, *MSH2*, *MCM10*, *RAD21* and *MDC1*), as represented in **Figure S7.** Therefore, we named subtype 3 the BL subtype.

#### Subtype 4: Suppressed (S) subtype

This subtype accounted for 15.8% of the TNBCs in the cohort. In contrast to the LAR, MLIA and BL subtypes, subtype 4 showed downregulation of multiple classic TNBC pathways, including immune-related pathways, cell growth, and cell apoptosis (**Figure 2E and 3A**). As shown in **Figure S7**, tumors in this subtype expressed decreased growth factor genes (*FGF2*, *IGF1*, *IGF2*, *IGFBP4*, *IGFBP7*, *PDGFRA*, *PDGFRB*, *PDGFC* and *PDGFD*), EMT-associated genes (*MMP2*, *ACTA2*, *SNAI2*, *SPARC*, *ZEB1*, *COL3A1*, *GNG11* and *ZEB2*), Wnt signaling genes (*CTNNB1*, *DKK2*, *SFRP4*, *TCF4*, *FZD4* and *CAV1*), angiogenesis genes (*KDR*, *TEK* and *EPAS1*), AR-driven genes (*AR*, *ALCAM*, *FKBP5*, *SPDEF*, *APOD*, *PIP* and *CLDN8*) and stem-like genes (*ABCA8*, *PROCR*, *ENG* and *THY1*). Due to the suppressed pathways and genes, we named subtype 4 the S subtype.

Then, we computed differentially expressed tandem 3′UTRs in each subtype by comparing the SUI of tandem 3′UTRs in one subtype with the others. Median SUI values were significantly differed between the four subtypes (ANOVA, F = 23.319, *p* < 0.001; **Figure S8**). Average median SUI was the highest in the BL subtype and the lowest in the S subtype. Comparing 3'UTR APA profile between the S subtype and the BL subtype, we identified 669 shortening tandem 3'UTRs (631 genes) and 644 lengthening tandem 3'UTRs (621 genes) with |ΔSUI|≥0.2 and FDR < 0.05 in the S subtype. Enrichment analysis of these genes were performed using Metascape and the results are shown in**Figure S9-10**. Differentially shortened tandem 3′UTRs in each subtype are shown in **Figure S11**. In the LAR subtype, the most shortened 3′UTR was *SERHL2*, which was associated with 4 mRNAs (**Figure S11A**). *RBP4* was significantly shortened in the MLIA subtype, and 38 mRNAs were tightly correlated with the tandem 3′UTR (**Figure S11B**). *PLEKHG4B* was highly shortened in the BL subtype, and the SUI of *PLEKHG4B* correlated with the expression of *PLEKHG4B* itself alone (**Figure S11C**). In the S subtype, *ITSN1* was remarkably shortened, and there was a positive correlation between the SUI of *ITSN1* and 3 mRNAs, including *ITSN1* gene itself (**Figure S11D**). Differential expression analyses indicated that these co-expressed mRNAs altered in the subtype to which the subtype-specific APA belongs (**Table S2**).

Next, we investigated the 479 tandem 3′UTRs of clinically actionable genes, including potential as well as FDA-approved drug targets and their associated genes (**Table S3**) 33-35. The percentage of tandem 3′UTRs that showed relatively large variances (interquartile range [IQR] ≥ 0.8) was 15.9%, 13.6%, 17.5% and 21.1% for LAR, MLIA, BL and S subtypes according to the FUSCC APA classification, respectively, and was 19.6%, 17.5%, 14.6%, 20.0%, 10.9%, 17.5% and 18.3% for BL1, BL2, IM, M, MSL, LAR and UNS subtypes based on Lehmann subtypes, respectively (**Figure 3B and C**). This suggests APA events may add an additional regulatory level to clinical actionable genes in TNBC.

Comparison of our FUSCC APA subtypes and Lehmann subtypes based on mRNA expression profiles showed that subtype 1 (LAR) contains 93.8% of all Lehmann LAR tumors and that subtype 3 (BL) contains 64.5% Lehmann BL1 and BL2 tumors, whereas subtype 2 (MLIA) is mainly composed of the Lehmann M, MSL and IM subtypes (**Figure S12**; **Table S4**).

### Clinical relevance of tandem 3′UTRs in TNBC

We investigated the clinicopathological characteristics of the 165 participants, who were predominantly (61.2%) postmenopausal with an average age of 53.5 years (standard deviation [SD] 10.8 years, median 54 years, range 27 to 83 years). All TNBCs were histologically confirmed invasive ductal carcinoma with no evidence of metastasis at diagnosis, and 63% of tumors were >2 cm at diagnosis. After a median follow-up time of 73.1 months, 35 of 165 patients experienced recurrence or death. As shown in **Table 1**, the APA subtype was associated with age at diagnosis, menopausal status, Ki-67, CK5/6, CK14 and Lehmann subtype (*p* < 0.05 for all). As shown in **Figure S13**, the highly proliferative nature of BL and S subtypes was supported by the high-intensity nuclear Ki-67 staining evaluated by immunohistochemistry analysis (mean Ki-67: LAR 38.8%, MLIA 47.2%, BL 57.7%, S 66.7%; *p* < 0.001, one-way ANOVA).

**Figure 4:**
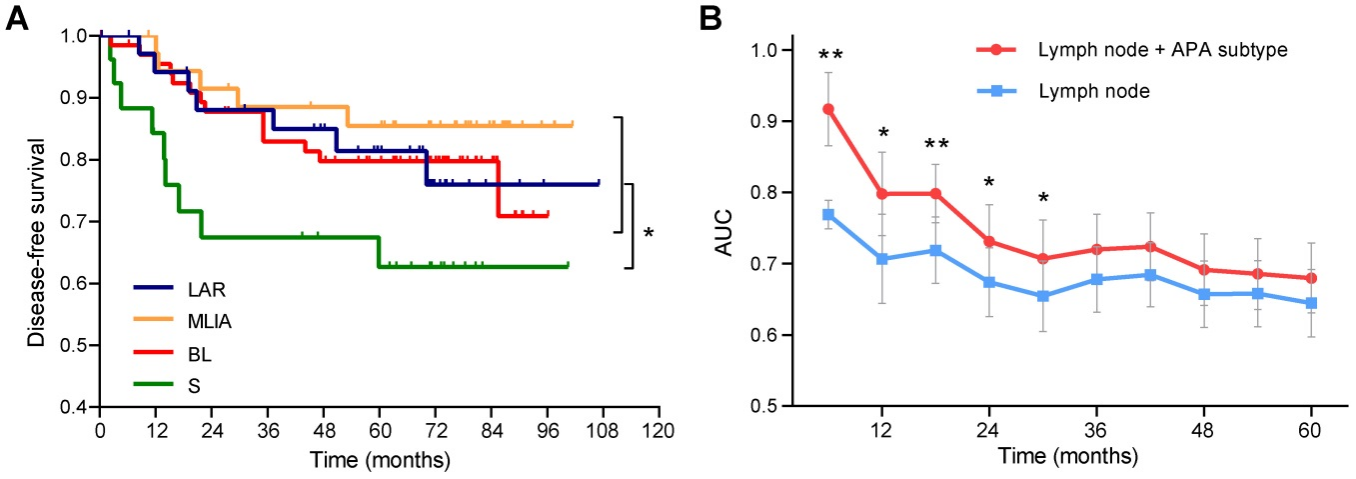
Prognostic significance of alternative polyadenylation subtype in triple-negative breast cancer. (**A**) Kaplan-Meier survival curves of disease-free survival (DFS) among the alternative polyadenylation (APA) subtypes. (**B**) Comparison of the area under the curve (AUC) of time-dependent receiver operating characteristic (ROC) curves with two Cox proportional-hazards models. blue: lymph node; red: lymph node + APA subtype. Data are shown as mean ± standard error. *p < 0.05, **p < 0.01. Abbreviations: APA, alternative polyadenylation; AUC, area under the curve; DFS, disease-free survival; ROC, receiver operating characteristic.

As shown in** Figure 4A**, patients in the S subtype had the worst disease-free survival (DFS) comparing to other patients (log-rank *p* = 0.021). Multivariate Cox analysis identified the S subtype (hazard ratio [HR] = 2.65, 95% confidence interval [CI]: 1.23-5.68, *p* = 0.013) and lymph node (HR = 2.99, 95% CI: 1.46-6.12, *p* = 0.0028) as independent prognostic factors (**Table 2**). Time-dependent ROC analysis indicated that the addition of APA subtype to the Cox proportional-hazards model significantly increased the prognostic efficacy of 6- (area under the curve [AUC]: 0.77* vs* 0.92, *p* = 0.0047), 12- (AUC: 0.71 *vs* 0.80, *p* = 0.034), 18- (AUC: 0.72* vs* 0.80, *p* = 0.0079), 24- (AUC: 0.67 *vs* 0.73, *p* = 0.019) and 30-month (AUC: 0.65 *vs* 0.71, *p* = 0.028) recurrence (**Figure 4B**).

### A pooled shRNA library screening identifies CPSF1 and PABPN1 as key cleavage and polyadenylation factors in TNBC

High proliferative activity is an important parameter of TNBC biology 2. Given the association of tandem 3'UTRs with cell proliferation 12, we speculated that modulating 3'UTR length might affect tumor progression. Cleavage and polyadenylation (C/P), resulting in different transcripts with different 3'UTR lengths, are regulated by 23 core *trans*-factors involving several single proteins and four multi-subunit protein complexes, which contain cleavage and polyadenylation specificity factor (CPSF), cleavage stimulation factor (CSTF), cleavage factor I (CFI) and cleavage factor II (CFII) 4, 36. Thus, we hypothesized that the aberrant expression of core C/P factors may lead to high proliferative tumor growth and dysregulated APA patterns. To address this hypothesis, we first investigated the gene expression of 23 core C/P factors within our microarray data. Twelve core C/P genes were more highly expressed (mean log_2_ fold change = 0.28; range 0.12 to 0.59) in TNBC tissues than in normal adjacent tissues (**Figure S14A**). The expression of the gene set exceeded that of the background set (*p* = 7.3 × 10^-7^; **Figure S14B**). The significantly up- and down-regulated core C/P factors between TNBC and matched normal adjacent tissues in each APA subtype are indicated by red and blue, respectively (**Figure S15**). The aberrant level of core C/P factors is expected to induce cleavage at inefficient proximal PASs, resulting in APA events. Thus, our data suggest that differential 3′-end processing could at least partially explain the APA events in TNBC.

**Figure 5:**
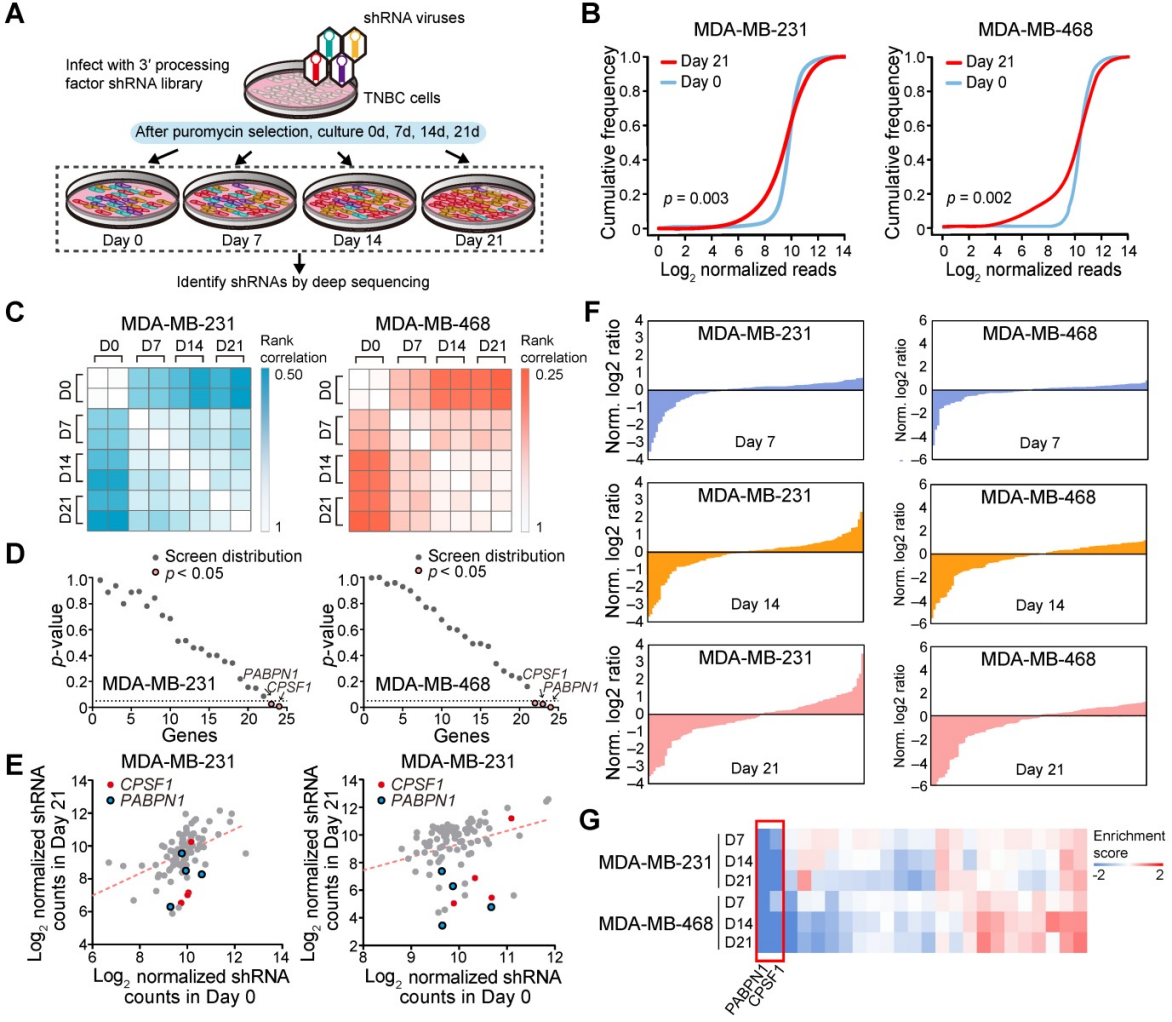
A pooled shRNA screen identifies *CPSF1* and *PABPN1* as key cleavage and polyadenylation factors in triple-negative breast cancer (TNBC). (**A**) Summary of the pooled shRNA screen in TNBC cell lines. (**B**) Cumulative frequency of shRNAs 0 and 21 days after transduction. The shift in the day 21 curve indicates the depletion in a subset of shRNAs. (**C**) Rank correlations of normalized read counts between biological replicates and culture days. Spearman's correlation coefficient was used for comparisons. (**D**) *P*-value for shRNA depletion in proliferation. (**E**) Scatterplot showing the depletion of specific shRNAs after 21 days of proliferation, highlighting *CPSF1* in red and *PABPN1* in blue. (**F**) Waterfall chart showing the enrichment of shRNAs in Day 7, 14 and 21 normalized to Day 0 read counts in MDA-MB-231 and MDA-MB-468 cells. For each cell line, the shRNAs were ranked on the basis of the mean normalized log_2_(Day 7/Day 0) ratios, log_2_(Day 14/Day 0) ratios and log_2_(Day 21/Day 0) ratios of the read counts. (**G**) The heatmap of enrichment score of each gene in two cell lines. Abbreviation: TNBC, triple-negative breast cancer.

Next, we used a proliferation-based shRNA library screening system to identify potential key 3'UTR modulatory factors in TNBC. Nearest shrunken centroids method identified MDA-MB-231 and MDA-MB-468 as BL subtype, the majority APA subtype, using tandem 3'UTR profiles. Thus, these two TNBC cell lines were chosen as a model in subsequent screening experiments. We screened 95 shRNAs targeting the 23 core C/P factors and 3 control hairpins in MDA-MB-231 and MDA-MB-468. After infecting the cells with lentiviruses expressing the shRNA library (MOI = 0.3) and subjecting them to selection, we continuously cultured the cells for 21 days and harvested cellular DNA for deep sequencing (**Figure 5A**). The abundance of each barcoded hairpin was quantified to identify shRNAs that were selectively depleted during cell proliferation. As expected, after 21 days a significant reduction in the diversity of shRNAs was observed in the surviving MDA-MB-231 and MDA-MB-468 cells (**Figure 5B**; *p* = 0.003 and 0.002, respectively). The read counts for most of the shRNA hairpins were consistent in two separate replicates of the proliferation assays (**Figure 5C**). As a result, the depletion of shRNA *CPSF1* and *PABPN1* was statistically significant after the 21-day proliferation assay in both MDA-MB-231 and MDA-MB-468 cells (*p* < 0.05; **Figure 5D-E**). **Figure S16** shows the number and percent of tandem 3′UTRs correlated with the 23 core C/P factors. *CPSF1* and *PABPN1* regulated a number of tandem 3′UTRs of clinically actionable genes. For example, the tandem 3′UTRs of *PIK3C2G*, *IL21A* and *RAD51D* were correlated with *CPSF1* mRNA expression (**Figure S17A**), whereas the tandem 3′UTRs of *NUDT5*, *HDAC7* and *HDAC1* were correlated with *PABPN1* mRNA expression (**Figure S17B**), which highlighted the potential drug targets of APA events in TNBC. We ranked the mean normalized read counts of the shRNAs identified in the proliferation assays on day 7, 14 and 21. A small number of shRNA hairpins were depleted in these screens, whereas most of the shRNAs showed few changes (**Figure 5F**; **Figure S18**). The enrichment scores for all shRNA hairpins in MDA-MB-231 and MDA-MB-468 are illustrated in **Figure 5G**. The hierarchical clustering heatmaps based on the enrichment score signature showed that the shRNAs were consistently changed in the replicates, indicating that shRNA library screening provides reliable results for identifying important C/P factors in TNBC.

### CPSF1 and PABPN1 knockdown reduces TNBC proliferation

**Figure 6:**
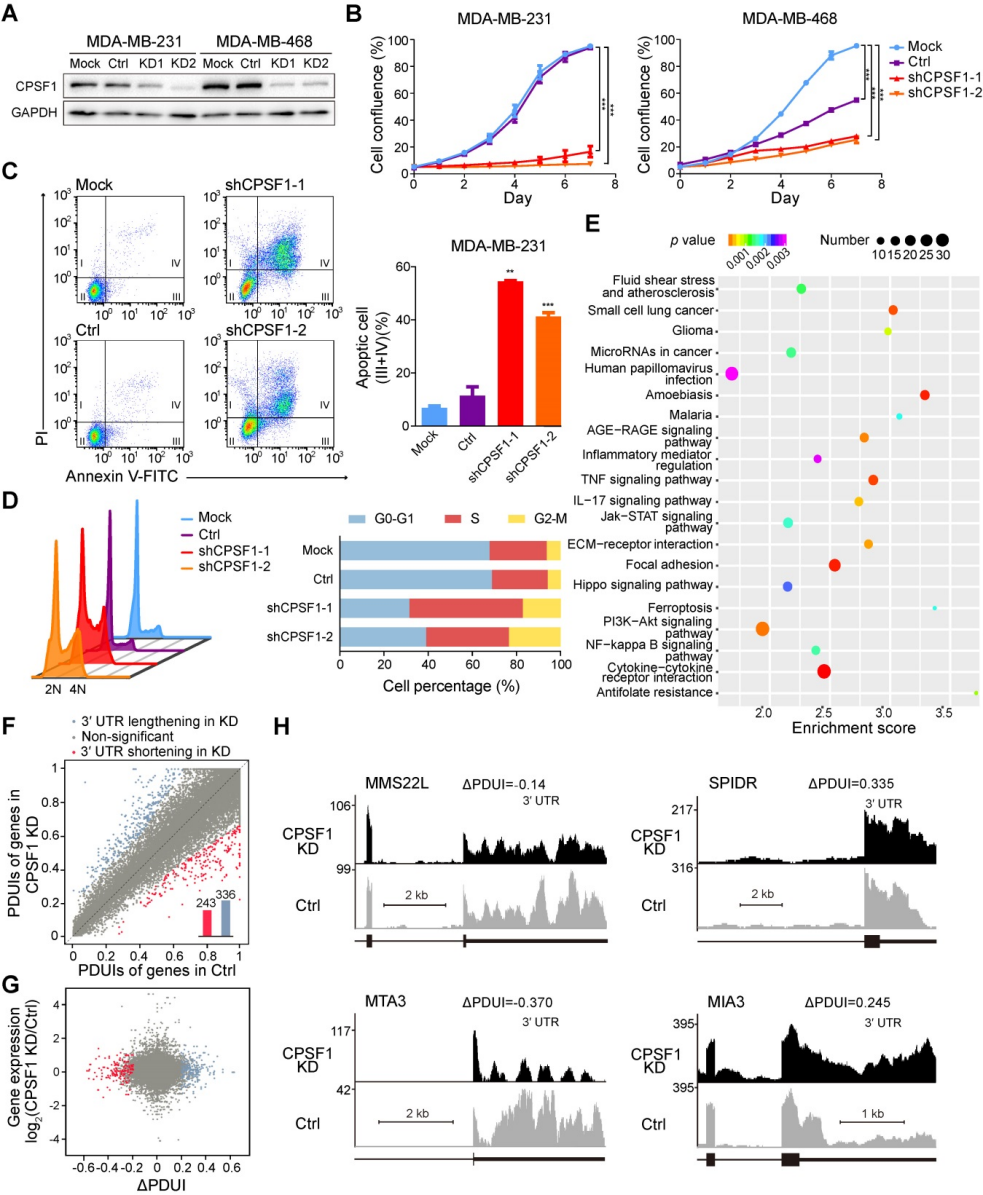
CPSF1 depletion results in decreased proliferation, enhanced apoptosis, cell cycle redistribution and alternative polyadenylation (APA) events. (**A**) Western blot analysis of MDA-MB-231 and MDA-MB-468 lysates infected with control and shRNA lentivirus targeting CPSF1. (**B**) Growth of MDA-MB-231 cells and MDA-MB-468 cells was measured after infection with CPSF1 shRNA lentivirus and control shRNA lentivirus. The results shown are the means ± standard deviation (s.d.) (*n* = 3). (**C**) CPSF1 knockdown increased the apoptosis rate in MDA-MB-231 cells (*n* = 3). (**D**) CPSF1 depletion led to cell cycle redistribution in MDA-MB-231 (*n* = 3). (**E**) Enriched pathways in CPSF1 knockdown MDA-MB-231 cells. (**F**) Scatterplot of percentage of distal poly(A) site usage index (PDUI) in control and CPSF1 knockdown MDA-MB-231 cells, where mRNAs were significantly shortened (*n* = 243) and lengthened (*n* = 336) after CPSF1 knockdown. (**G**) Correlation between distal poly(A) site usage and gene expression levels of control and CPSF1 knockdown MDA-MB-231 cells. (**H**) Representative RNA-seq density plots along with ΔPDUI values for genes whose 3′UTR was unchanged (*MMS22L*), lengthened (*SPIDR* and *MIA3*) and shortened (*MTA3*) in response to CPSF1 knockdown. **p < 0.01, ***p < 0.001. Abbreviations: APA, alternative polyadenylation; PDUI, percentage of distal poly(A) site usage index.

Because TNBC is a highly proliferative cancer, we reasoned that TNBC cell lines might be sensitive to changes in the CPSF1 and PABPN1 levels. To validate the screening results stated above, we assessed the effects of CPSF1 and PABPN1 knockdown in MDA-MB-231 and MDA-MB-468 cells. We successfully constructed stable cell lines with CPSF1-knockdown using lentivirus shRNA infection (**Figure 6A**). As expected, the proliferation of CPSF1-knockdown cells was hampered relative to the proliferation of control knockdown cells (**Figure 6B**). Consistent with this observation, the apoptosis level was increased in CPSF1-knockdown TNBC cells (**Figure 6C**;**Figure S19A**). We found that knockdown of CPSF1 induced cell cycle arrest at the S phase in MDA-MB-231 (**Figure 6D**) and MDA-MB-468 (**Figure S19B**). Moreover, an increase in the percentage of cells in G2/M phase was observed in CPSF1-depleted MDA-MB-231 cells (**Figure 6D**). These results indicate that CPSF1 depletion leads to weakened cell proliferation and increased rates of apoptosis and cell cycle redistribution, which supports the hypothesis that *CPSF1* is a novel pro-proliferative gene whose levels may be enhanced in human cancer.

To further identify the targets of CPSF1 in TNBC, we performed RNA sequencing (RNA-seq) with a depth of > 1 × 10^8^ reads after knocking down CPSF1 in parallel with a control knockdown. Differential expression genes were determined, and 356 up-regulated genes and 501 down-regulated genes were identified in CPSF1-knockdown cells (**Figure S20**). Pathway analysis revealed that several classic TNBC pathways, including the JAK/STAT signaling pathway, ECM receptor pathway and PI3K/Akt pathway, were affected in CPSF1-knockdown cell lines (**Figure 6E**). Then, we used the well-established algorithm DaPars to examine the 3'UTR alterations between control and CPSF1 knockdown cells. DaPars identified 579 transcripts possessing a significant shift in 3'UTR usage in response to CPSF1 depletion (**Figure 6F**). However, there was no statistically significant correlation between APA events and gene expression in CPSF-knockdown cells (**Figure 6G**). We observed 21 initial 3'UTR shortening or lengthening events in TNBC samples were reversed in CPSF1-depleted TNBC cells (**Figure S21 A-B**). **Figure 6H** shows several genes (*MMS22L*, *SPIDR*, *MTA3* and *MIA3*) whose APAs were altered by CPSF1 knockdown. We observed not only shortening of 3'UTRs, but also lengthening and no changes, indicating that the CPSF complex regulates many, but not all, genes capable of APA. In TCGA cohort, the 3'UTR shortening of *SPIDR* was associated with poor survival whereas no survival difference was observed when patients were divided by APA events of *MTA3* or *MIA3* (**Figure S22**).

PABPN1 has been proven to be the master factor regulating the APA profile across multiple cancer types 37. Using the pooled shRNA library screening system, we re-identified PABPN1 as a key regulator in TNBC. The PABPN1-knockdown stable cell lines showed lower proliferation rate than control TNBC cells (**Figure S23**). Enhanced apoptosis was observed in PABPN1-depleted MDA-MB-231 and MDA-MB-468 cells (**Figure S24**). PABPN1 knockdown also resulted in S phase arrest in MDA-MB-468 cells, but this phenotype was not verified in MDA-MB-231 cells (**Figure S25**), whereas an accumulation of cells at the G2/M phase was observed in PABPN1-depleted MDA-MB-231 cells (**Figure S25A**). RNA-seq analysis revealed 789 up-regulated genes and 640 down-regulated genes in PABPN1-depleted cells (**Figure S26**). The altered genes were enriched in BL characteristic pathways (cell cycle, DNA replication) and LAR classic pathways (tryptophan metabolism, tyrosine metabolism) (**Figure S27**). DaPars identified 858 shortened 3′UTRs and 321 lengthened 3′UTRs in PABPN1-knockdown cells (**Figure S28A**). No correlation between APA events and gene expression in PABPN1-knockdown cells was observed (**Figure S28B**). Forty initial 3'UTR shortening or lengthening events were reversed in PABPN1-depleted TNBC cells (**Figure S21C-D**). Five genes (*SLC9A1*, *SPIDR*, *ZFP62*, *OSGEPL1* and *NUCB2*) displayed 3'UTR shortening in TNBC samples and lengthened in both CPSF1- and PABPN1-knockdown TNBC cells. SLC9A1 is a promising drug target of TNBC. It encodes a major pH regulatory protein, which facilitates metastasis of TNBC. Preclinical studies have shown pharmacological inhibition of SLC9A1 can amplify the anti-cancer effects of paclitaxel in MDA-MB-231 and MD-MB-468 cells 38. NUCB2 is a potential biomarker for breast cancer metastasis 39. There, focusing on the APA regulation in TNBC may provide new insights for designing novel targeted drug therapies. **Figure S28C** illustrates the 3'UTR alterations of multiple genes (shortening: *MMS22L*, *DHX36* and *MUM1*; lengthening: *PHF21A*) after PABPN1 knockdown. However, there was no survival difference when patients were dichotomized according to the APA events of *DHX36*, *PHF21A* and *MUM1* (**Figure S29C**). Collectively, CPSF1 and PABPN1 knockdown recapitulates changes in APA patterns and retards proliferation in TNBC.

## Discussion

The emerging role of 3'UTR APA in carcinogenesis has provided a new approach for multilayer analysis of cancer. With the large-scale microarray transcriptome data from our center, we provided a comprehensive view of the APA landscape in TNBC. In this study, we developed a Bayes-based strategy to study the APA events in a large TNBC cohort using transcriptome microarray technology. With the NMF method, TNBCs were classified into four stable subtypes (LAR, MLIA, BL and S) according to the tandem 3'UTR signature. These findings highlight the molecular heterogeneity and complexity of the APA profiles in TNBCs. Furthermore, a pooled shRNA library screening identified *CPSF1* and *PABPN1*, two alternative C/P factors, as potential targets of TNBC linking APA to tumor proliferation. To our knowledge, this study is the first to profile the 3'UTR landscape using transcriptome microarrays in a large TNBC cohort with well-recorded clinical annotations, leading to the identification of a novel potential therapeutic target.

The 3'UTR length is known to undergo dynamic changes under malignant conditions in diverse physiological and pathological conditions. We focused our study on TNBC, because this subtype of highly heterogeneous breast cancer possesses high proliferative indices and often presents an aggressive clinical course. Recent studies identified multiple 3'UTRs displaying length preferences in TNBC compared with non-TNBC or adjacent breast tissue 32, 40, 41. Furthermore, a probe-based meta-analysis using 3'-based chips reported shorter 3'UTRs in TNBC 32. A mechanistic study reported that APA in TNBC allows NRAS and c-JUN to bypass PUMILIO post-transcriptional regulation 42. Conversely, deep-sequencing of cell lines revealed preferential lengthening of the 3'UTRs in TNBC tissues compared with luminal breast cancers and normal breast tissues 41. Several small-scale studies recently investigated the APA landscape of breast cancer. Kim and colleagues dissected APA patterns using RNA sequencing from 515 single cells from 11 patients with breast cancer and identified cell type specific APA 43. Besides, a modified polyadenylation site sequencing (PAS-seq) , direct capture of mRNA 3' ends , was used to characterize the APA events in 12 pairs of HER2-negative breast cancer and adjacent normal samples (six ER-positive and 6 ER-negative cancers) 44. Gillen *et al.* revealed the APA event in *PRELID1* is a strong subtype-dependent predictor of breast cancer patients clinical outcomes 44. Fu *et al.* profiled APA sites in two breast cancer cell lines (MCF7 and MDA-MB-231) and one cultured mammary epithelial cell line (MCF10A) using sequencing APA sites (SAPAS) method and demonstrated the genes with APA events and the enriched pathways, including cell cycle, apoptosis and metabolism 41. However, conventional APA characterization has not been extensively adopted, and the large-scale data of global APA events in a specific cancer subtype are relatively limited. We previously established the prognostic value of both 3'UTR shortening and lengthening in TNBC 40. Our previous study also proved that the 3'UTR-based model could predict nodal status in TNBC 45. Here, using transcriptome arrays, we determined that the direction of 3'UTR length changes varies in genes, although more than half of the tandem 3'UTRs prefer shortening isoforms. In addition, the multimodal correlation between gene expression and the 3'UTR length contributes to transcriptome complexity and provides an indispensable layer of gene regulation.

Considering the emerging roles of APA in carcinogenesis, we subtyped TNBC using APA profiles to systematically understand the heterogeneous nature of TNBC. Intriguingly, compared to the Lehmann system generated from mRNA expression profile 15, the APA subtypes had considerable overlaps between the two systems. For example, APA subtype 1 (LAR) contains 93.8% of Lehmann LAR cancers. APA subtype 3 (BL), characterized by distinct basal properties, is enriched in cell cycle and cell division related pathways. Moreover, the BL subtype showed high Ki-67 staining, supporting its highly proliferative nature. Using a lncRNA-mRNA comprehensive signature, we previously demonstrated that cell division and cell cycle related pathways were enriched in the basal-like and immune-suppressed (BLIS) subtype (FUSCC classification)^17^. Most recently, we have re-identifed BLIS subtype using genomic and transcriptomic landscape of TNBC 46. Burstein and his colleagues 16 also proposed four TNBC subtypes based on RNA and DNA profiles, including two basal-like clusters as well. Thus, the TNBC subgroups identified on the basis of APA signatures reflect the intrinsic phenotypes with different pathway features. In addition, the 3'UTR length of cell cycle-related genes is dysregulated in both normal tissue (testis and embryonic stem cells) 23 and cancerous lesions (colorectal cancer) 30, which suggests that the 3'UTR length of cell cycle genes is associated with the pathway activation status.

Purification of 3' processing complexes and subsequent proteomic and structural characterization identified over 80 proteins, of which ~ 20 are core 3' processing factors, which consists of four protein complexes (CPSF, CSTF, CFI and CFII) and several single proteins, including symplekin, retinoblastoma-binding protein 6 (RBBP6) 4, 36, 47. PABPN1 might also be considered to be a core factor 4. It disrupts the interaction between CPSF and the poly(A) polymerase via binding to the growing poly(A) tail when the tail is ~250 nucleotides, and thus the poly(A) tail length is controlled 48-50. In this study, we performed shRNA pooled library screening and identified *CPSF1* and *PABPN1* as key C/P factors involved in tumor proliferation. *PABPN1* has been proven to be the master factor regulating APA profiles across multiple cancer types in a large TCGA cohort 37, which supports the efficacy of our proliferation screening system. PABPN1 regulates APA by preventing the usage of weak proximal PASs 51. PABPN1, together with CPSF and poly(A) polymerase (PAP), is required for efficient polyadenylation *in vitro* 49, 50. CPSF1 (CPSF-160) recognizes the polyadenylation signal (most frequently AAUAAA) and is a key member of CPSF, a multisubunit complex that plays a central role of the polyadenylation machinery in metazoans 52. Recent studies independently observed the global up-regulation of genes encoding 3' processing factors in cancer cells 13, 30, 31. *CPSF1* and *CSTF2* display the most striking differences in expression 13. NUDT21 inhibits bladder cancer progression by regulation of APA-mediated 3'UTR alterations 53. These data suggest that the regulation of APA by altering the levels of core processing factors (especially *CPSF1* and* PABPN1*) may be a general mechanism. Significantly, the data extend our understanding of APA in regulated gene expression and pathway activation by demonstrating that extensive 3'UTR shortening is enriched in cell cycle genes, and that the intervention of APA regulation inhibits cell proliferation in TNBC. Intriguingly, we observed a reversion of APA events occurred after CPSF1 or PABPN1 knockdown. The involved genes participate in drug resistance (*SCL9A1* 38, *CCSAP* 54, *NUP98* 55, *PLD1* 56), tumorigenesis (*DEPDC5* 57), proliferation (*MED15* 58, *STX3* 59) and metastasis (*ARHGEF26* 60, *ATP6V0A2* 61, *MBD4* 62) in breast cancer and are breast cancer biomarkers (*ITPR1*^63^, *NUP98* 55, *GATA3* 64, 65, *DOCK4* 66, *OSR1* 67) and potential drug targets for TNBC (*SCL9A1* 38, *LRP8* 68, 69). It suggests understanding APA regulation mediated by the core 3' processing factors, especially the target specificity and directionality, may provide new ideas for targeted therapy in TNBC.

We profiled the APA events of TNBC using publicly available HG-U133 series microarrays before 40, 45. However, 3'-based chips severely limit the number of genes, and only 1,933 (~9.7%) of human protein-coding genes were investigated. Here, we provide a less biased 3'UTR landscape of TNBC using transcriptome arrays with high probe intensity. We applied Bayesian change point analysis rather than a modified *t* test to individuals. The multivariate Bayesian approach estimated the probes' posterior probabilities and the posterior means simultaneously, which substantially reduced the number of statistical calculations. Next-generation sequencing has also been used for 3'UTR analysis with other bioinformatics algorithms, including DaPars 70, SAPAS 41, and 3'-Seq 30. However, complicated library preparation procedures and the high expenditure for deep sequencing depth (×1000 or more for some algorithms) limit the use for large cohort profiling and analyses. The Bayes-based microarray analysis strategy balances measuring accuracy and economy and is suitable for large-scale detection of APA events.

Our study has several limitations. First, transcriptome microarray data limited the analyses at the level of the transcript. We investigated correlation patterns between 3'UTR length and mRNA expression, rather than protein level, which constitutes a primary limitation of this study. Second, because probe-intensity-based analysis is limited by the original chip design, the APA-based 3'UTR length alterations we report here are likely to under-represent the actual complexity. Compared with RNA-seq, HTA 2.0 cannot identify novel APAs. Future experiments with RNA-seq or expanded mRNA probe sets paired with protein arrays would therefore help to confirm and expand our findings. Third, due to the limited data on tandem 3'UTRs in TNBC, the new subtyping system has not been validated in other independent cohorts. In addition, the follow-up time of our cohort is relatively short. In spite of the large computational effort, our analysis was the first step to understand the APA regulations across different TNBC subtypes. Therefore, our future work will focus on recruiting independent TNBC cohorts to validate the APA classifier, updating the follow-up data and investigating the functions of novel APA events in each subtype.

Collectively, we profiled the APA landscape in TNBC and developed a novel TNBC subtyping system, assigning patients with TNBC to four distinct subtypes based on the APA signature. In addition, we established *CPSF1* and *PABPN1* as key C/P factors involved in tumor proliferation and APA regulation in TNBC. Once further validated in a larger population, the APA classification system could improve personalized treatment of TNBC.

## Supplementary Material

Supplementary methods and figures.

Supplementary tables.

## Figures and Tables

**Table 1:** Clinicopathological characteristics of the alternative polyadenylation (APA) subtypes of TNBC

Characteristics	Number of patients (%)	FUSCC APA subtypes				*p*-value
		LAR	MLIA	BL	S	
		*n* (%)	*n* (%)	*n* (%)	*n* (%)	
Total	165	36	36	67	26	
**Age**						**0.001**
≤ 50 years	68 (41.2)	7 (19.4)	10 (27.8)	38 (56.7)	13 (50.0)	
> 50 years	97 (58.8)	29 (80.6)	26 (72.2)	29 (43.3)	13 (50.0)	
**Menopausal status**						**0.006**
Premenopause	64 (38.8)	7 (19.4)	12 (33.3)	36 (53.7)	9 (34.6)	
Postmenopause	101 (61.2)	29 (80.6)	24 (66.7)	31 (46.3)	17 (65.4)	
**Tumor size**						0.370
≤ 2 cm	58 (35.2)	14 (38.9)	12 (33.3)	21 (31.3)	11 (42.3)	
> 2 cm	104 (63.0)	22 (61.1)	24 (66.7)	45 (67.2)	13 (50.0)	
Unknown	3 (1.8)	0 (0.0)	0 (0.0)	1 (1.5)	2 (7.7)	
**Lymph node**						0.336
Negative	86 (52.1)	14 (38.9)	19 (52.8)	38 (56.7)	15 (57.7)	
Positive	79 (47.9)	22 (61.1)	17 (47.2)	29 (43.3)	11 (42.3)	
**Grade**						0.071
I-II	32 (19.4)	12 (33.3)	9 (25.0)	10 (14.9)	1 (3.8)	
III	104 (63.0)	20 (55.6)	19 (52.8)	46 (68.7)	19 (73.1)	
Unknown	29 (17.6)	4 (11.1)	8 (22.2)	11 (16.4)	6 (23.1)	
**Ki-67**						**0.042**
< 30%	32 (19.4)	12 (33.3)	8 (22.2)	10 (14.9)	2 (7.7)	
≥ 30%	132 (80.0)	23 (63.9)	28 (77.8)	57 (85.1)	24 (92.3)	
Unknown	1 (0.6)	1 (2.8)	0 (0.0)	0 (0.0)	0 (0.0)	
**Neurovascular invasion**						0.551
Negative	70 (42.4)	13 (36.1)	13 (36.1)	32 (47.8)	12 (46.2)	
Positive	95 (57.6)	23 (63.9)	23 (63.9)	35 (52.2)	14 (53.8)	
**EGFR**						0.067
Negative	107 (64.8)	17 (47.2)	24 (66.7)	49 (73.1)	17 (65.4)	
Positive	54 (32.7)	18 (50.0)	12 (33.3)	17 (25.4)	7 (26.9)	
Unknown	4 (2.4)	1 (2.8)	0 (0.0)	1 (1.5)	2 (7.7)	
**CK5/6**						**0.002**
Negative	72 (43.6)	20 (55.6)	20 (55.6)	28 (41.8)	4 (15.4)	
Positive	90 (54.5)	15 (41.7)	16 (44.4)	39 (58.2)	20 (76.9)	
Unknown	3 (1.8)	1 (2.8)	0 (0.0)	0 (0.0)	2 (7.7)	
**CK14**						**<0.001**
Negative	94 (57.0)	31 (86.1)	26 (72.2)	29 (43.3)	8 (30.8)	
Positive	68 (41.2)	4 (11.1)	10 (27.8)	38 (56.7)	16 (61.5)	
Unknown	3 (1.8)	1 (2.8)	0 (0.0)	0 (0.0)	2 (7.7)	

**Abbrevations:** BL: basal-like; CK: cytokeratin; EGFR: epidermal growth factor receptor; LAR: luminal androgen receptor; MLIA: mesenchymal-like immune-activated; S: suppressed.

**Table 2:** Univariate and multivariate Cox proportional hazard model for disease-free survival

Variables	Univariate		Multivariate	
	HR (95% CI)	*p*-value	HR (95% CI)	*p*-value
Age (> 50 years *vs* ≤ 50 years)	0.61 (0.31-1.18)	0.14	-	-
Tumor size (> 2 cm *vs* ≤ 2 cm)	1.41 (0.67-2.97)	0.36	-	-
Lymph node (positive *vs* negative)	2.81 (1.37-5.75)	0.0046	2.99 (1.46-6.12)	0.0028
Ki-67 (≥ 30% *vs* < 30%)	1.03 (0.45-2.37)	0.94	-	-
APA subtype (S *vs* others)	2.37 (1.11-5.08)	0.026	2.65 (1.23-5.68)	0.013

**Abbrevations:** APA: alternative polyadenylation; CI: confidence interval; HR: hazard ratio; S: suppressed.

## Abbreviations

3'UTR: 3' untranslated region
ANOVA: analysis of variance
APA: alternative polyadenylation
AUC: area under the curve
BCP: Bayesian analysis of the change point
BL: basal-like
CFI: cleavage factor I
CFII: cleavage factor II
CI: confidence interval
C/P: cleavage and polyadenylation
CPSF: cleavage and polyadenylation specificity factor
CSTF: cleavage stimulation factor
CV: coefficient of variation
DFS: disease-free survival
ECM: extracellular matrix
ER: estrogen receptor
ES: enrichment score
FDR: false discovery rate
FUSCC: Fudan University Shanghai Cancer Center
GEO: Gene Expression Omnibus
HER2: human epidermal growth factor receptor 2
HR: hazard ratio
HTA 2.0: Human Transcriptome Array 2.0
LAR: luminal androgen receptor
MLIA: mesenchymal-like immune-activated
MOI: multiplicity of infection
NES: normalized enrichment score
NMF: non-negative matrix factorization
nsNMF: non-smooth NMF
PAP: poly(A) polymerase
PAS: poly(A) site
PCA: principal component analysis
PDGF: platelet-derived growth factor
PDUI: percentage of distal poly(A) site usage index
PPAR: peroxisome proliferator-activated receptor
PR: progesterone receptor
RBBP6: retinoblastoma-binding protein 6
ROC: receiver operating characteristic
RPPA: reverse phase protein arrays
S: suppressed
SD: standard deviation
shRNA: short hairpin RNA
SUI: short 3′UTR index
TCGA: The Cancer Genome Atlas
TNBC: triple-negative breast cancer
