# Supplementary material for: Dissecting the heterogeneity of the alternative polyadenylation profiles in triple-negative breast cancers
Source: Theranostics. 2020 Aug 21;10(23):10531–47. doi: 10.7150/thno.40944 (PMC7482814; doi:10.7150/thno.40944)
Supplement: Supplementary file 1 — Supplementary methods and figures. [file thnov10p10531s1.pdf]

SUPPLEMENTARY DATA

Dissecting the heterogeneity of the alternative polyadenylation profiles in triple-negative breast cancers

SUPPLEMENTARY METHODS

Transcriptome array data analysis

We processed the CEL files with aroma.affymetrix [1] using robust microarray analysis (RMA) background correction and quantile normalization. The probe intensities were extracted from the intermediate CEL files for probe-level analysis and log2 transformed. We then normalized the probe intensities to the gene expression level. Gene expression was profiled using the custom CDF from Brainarray (http://brainarray.mbni.med.umich.edu/Brainarray/Database/CustomCDF/).

Pathway and co-expression network analyses

Each TNBC subtype was computed for gene enrichment compared with all other tumor samples using gene set enrichment analysis (GSEA) software [2]. Genes were examined for enrichment in the C2 curated gene sets of canonical pathways. With the GSEA algorithm (1,000 permutations), the top significantly enriched canonical pathways were selected based on a normalized enrichment score (NES) greater than 0.4 and a false discovery rate (FDR) q value of less than 0.60 [3]. Metascape was used to identify pathway enrichment in genes with APA events [4]. Pathway or process terms with minimum count of 3, p-value<0.01, and enrichment factor>1.5 were considered significant.

To investigate interactions between tandem 3′UTRs and mRNAs, we constructed co-expression networks[5]. We screened for differentially expressed tandem 3′UTRs and mRNAs among subtypes. We computed the Pearson correlation and chose pairs (only 3′UTR-mRNA) with significant correlation to build the network (adjusted $p < 0.05$). Only those with Pearson correlation coefficient $\gamma > 0.75$ or $\gamma < -0.75$ are represented. The co-expression networks were visualized using Cytoscape 2.8.2 [6]. The Bioconductor package ‘limma’ [7] was used to examine differential expression of co-expressed mRNAs.

Pooled shRNA screening

pLKO.1 lentiviral plasmids encoding short hairpin RNAs (shRNAs) targeting 3' processing factors and nontargeting controls were each used as a pool. shRNA lentiviruses were designed according to the information in the RNAi Consortium (Broad Institute of MIT and Harvard) and generated from HEK293T cells. MDA-MB-231 and MDA-MB-468 cells were infected with lentiviral supernatant containing shRNAs with a multiplicity of infection (MOI) of 0.3 to ensure that each cell contained only one viral integrant. After 48 hours, the cell medium was replaced with medium containing 1 μg/ml puromycin. After cells were selected for 72 hours with puromycin, a minimum of 2×10^7 cells were harvested (Day 0) and the remaining cells were split into triplicate flasks (at least 2×10^7 cells per flask) and cultured for an additional 7, 14 and 21 days before genomic DNA extraction and analysis, respectively. Cells were collected to obtain genomic DNA. The shRNAs encoded in the genomic DNA were amplified, and adaptors with indexes for deep sequencing were incorporated into PCR primers. Sample quantification was performed on a Qubit fluorometer (Life Technologies) to ensure that samples were pooled at the same quantity. Deep sequencing was performed using the

MiSeq Personal Sequencer (Illumina). shRNA barcodes were retrieved and deconvoluted from each sequencing read. Then, the number of reads for each unique shRNA for a given sample was normalized as follows:

$$normalized\ reads\ per\ shRNA = \frac{reads\ per\ shRNA}{total\ reads\ for\ all\ shRNA\ in\ sample} \times 10^5 + 1$$

For each gene *G* with *k* barcodes (shRNA), each with average shRNA count *c* in the Day 0 group and *d* in the Day 7, 14 or 21 group, an enrichment score (ES) was computed as the second lowest ranked value of

$$t_i(G) = \log_2\left(\frac{d_i(G)}{c_i(G)}\right), i \text{ in } (1, k)$$

Subsequently, the *p*-value for each gene *G* was computed on the basis of ES as

$$P(G) = \frac{1}{N}\sum_{i=1}^{N} q_i$$

where

$$q_i = \begin{cases} 0 \text{ if } s_i(k) > ES \\ 1 \text{ if } s_i(k) \leq ES \end{cases}$$

and *s*(*k*) was the second lowest ranked value of *k* randomly chosen values from all barcodes in all genes and *N* was the number of permutation trials performed. *N* was set at 10,000.

Cell culture

The human breast cancer cell lines MDA-MB-231 and MDA-MB-468 and the human embryonic kidney cell line HEK293T were obtained from the Shanghai Cell Bank Type Culture Collection Committee (CBTCCC, Shanghai, China) and cultured in DMEM with 10% fetal bovine serum (FBS). The identities of the cell lines were confirmed by the CBTCCC using DNA profiling (short tandem repeat, STR). All cell lines were maintained in our laboratory and subjected to routine quality control (e.g., mycoplasma, morphology) by HD Biosciences every 3 months. Cells were

passaged for less than 6 months.

Stable cell line construction

pLKO.1 lentiviral plasmids encoding shRNAs targeting *CPSF1* and *PABPN1* and nontargeting control were used. We used the following shRNA sequences: CPSF1 shRNA1: 5'-GCTACTTCGAGGATATTTA-3'; shRNA2: 5'-CGGGTTTGTGCAGAATGTA-3'; PABPN1 shRNA1: 5'-GTAGAGAAGCAGATGAATA-3'; PABPN1 shRNA2: 5'-GGTAGAGAAGCAGATGAAT-3'; control shRNA: 5'-TTCTCCGAACGTGTCACGT-3'. All shRNA constructs were purchased from Genechem (Genechem Co., Shanghai, China). HEK293T cells were co-transfected with vector plasmid and packaging plasmids using polyethylenimine. Viral supernatants were harvested 48 hours later and stored at -80°C. MDA-MB-231 and MDA-MB-468 cells were infected with lentiviral supernatant for 48 hours. Then, the cells were selected for 5 days with puromycin (1 μg/ml) for subsequent use. Untreated cells were used as "mock" to provide a reference for the treated cell.

Western blotting

Whole-cell extracts were obtained using SDS lysis buffer (Beyotime) with protease and phosphatase inhibitors (Bimake). The cell lysates were boiled in 5× SDS-PAGE loading buffer for 5 minutes. Then, the proteins were separated by SDS-PAGE and transferred to polyvinylidene difluoride membranes (Roche). The membranes were blocked for 60 minutes with 5% skim milk in TBST and blotted with the following primary antibodies for 12-16 hours at 4 °C: rabbit polyclonal anti-CPSF1 antibody (Bethyl; 1:2,000, catalog no. A301-508A), rabbit monoclonal anti-PABPN1 antibody

(Abcam; 1:2,500, catalog no. EP3000Y), and mouse monoclonal anti-GAPDH (ProteinTech; 1:5,000, catalog no. 60004-1-lg). After extensive washing with TBST, the membranes were incubated for 60 minutes at room temperature with HRP-conjugated goat anti-rabbit antibody (Jackson ImmunoResearch; 1:5,000) or goat anti-mouse antibody (Jackson ImmunoResearch; 1:5,000), and signals were detected with and enhanced chemiluminescence substrate (Pierce Biotechnology). We used Molecular Imager ChemiDoc XRS+ with Image Lab Software (Bio-Rad) to acquire the images.

Cell proliferation

The cells of interest (2×10^3 cells per well) were seeded in 96-well, clear-bottomed plates with 100 μl of complete culture medium for 7 days. The IncuCyte® ZOOM Live-Cell Analysis System (Essen) was used to monitor proliferation and determine cell confluence.

Apoptosis assay and cell cycle analyses

Cell apoptosis was assessed using the FITC Annexin V Apoptosis Detection Kit I (BD Pharmingen) followed by flow cytometry (FACStation, BD Biosciences) according to the manual. For the cell cycle assay, cells were stained with propidium iodide (Beyotime) and analyzed by flow cytometry as described [8].

RNA-seq data analysis

Total RNA was purified using TRIzol reagent (Invitrogen). RNA integrity was evaluated using Agilent 2100 Bioanalyzer. Samples with an RNA Integrity Number (RIN) greater than 9 were used

for cDNA library construction. RNA-seq libraries were constructed using the TruSeq Stranded mRNA LTSample Prep Kit (Illumina) according to the manufacturer's instructions. The paired-end reads with 150 nt at each end, sequenced using the Illumina HiSeq X-Ten platform, were aligned to the human genome (hg19) using HISAT2 [9]. The fragments per kilobase of transcript sequence per million mapped paired-end reads (FPKM) value of each gene was calculated using cufflinks [10]. Differentially expressed genes (DEGs) were computed using the Bioconductor package 'DESeq' (version 3.8). *P*-value < 0.05 and fold change > 2 or < 0.5 were set as the threshold for significantly different expression. KEGG pathway analysis [11-13] of DEGs was performed using R based on hypergeometric distribution.

Profiling APA events from RNA-seq data

We used the well-established algorithm 'dynamic analysis of alternative polyadenylation from RNA-seq' (DaPars) (https//github.com/ZhengXia/DaPars) [14, 15] to identify and quantify dynamic APA events between control and core 3' processing factors depleted cell lines. The percentage of Distal polyA site Usage Index (PDUI) for each transcript was computed. Genomic coordinates of RNA transcripts from UCSC genome browser (hg19) were used to compute APA events from RNA-seq data. To avoid false-positive estimation of low abundance genes, we included transcripts whose coverage of last exon is more than 30-fold in at least one sample in each group in the subsequent analysis as suggested by DaPars. For characterization of the APA events in two groups, mean PDUIs were computed and Fisher's exact test was used to compare the difference of PDUIs between two groups. *P*-values were adjusted by Benjamini-Hochberg (BH) procedures, and the false-discovery rate (FDR) was reported. The following criteria were used to detect significant APA events: (1) The

FDR was controlled at 0.05 level. (2) The absolute mean difference of PDUIs between the two groups should be no less than 0.2. (3) The mean log2-fold-change of PDUIs in the knockdown (KD) group must be more than 0.59 (fold-change ≥ 1.5).

$$\begin{cases} \mathrm{FDR} \leq 0.05 \\ |\Delta \mathrm{PDUI}| = |\mathrm{PDUI}_{\mathrm{KD}} - \mathrm{PDUI}_{\mathrm{ctrl}}| \geq 0.2 \\ \left|\log_2\left(\frac{\mathrm{PDUI}_{\mathrm{KD}}}{\mathrm{PDUI}_{\mathrm{ctrl}}}\right)\right| \geq 0.59 \end{cases}$$

The PDUI data of breast cancer samples from the Cancer Genome Atlas (TCGA) were downloaded from TC3A.org (http://www.tc3a.org/) [16]. Feng and colleagues [16] downloaded RNA-seq BAM files of 10,537 cancer samples across 32 TCGA cancer types from the UCSC Cancer Genomic Hub (CGHub). DaPars were used to compute the PDUI. The other TCGA data were downloaded from the TCGA Data Coordination Center (DCC) or from the the results of the TCGA Firehose pieline at the Broad Institute (http://gdac.broadinstitute.org/).

Statistical analysis

The Pearson χ^2 test was used to compare qualitative variables, and Fisher's exact test was performed when necessary. We used linear models for microarray and RNA-seq data (LIMMA) [17, 18] to perform the differential expression analysis for microarray data. The Kolmogorov-Smirnov test was used to compare the cumulative distribution between two samples. Disease-free survival (DFS) was calculated from the date of surgery to the date of disease relapse at a local, regional or distant site, or patient death. Overall survival (OS) was defined as the time from the date of surgery to the date of patient death. Patients with a study end date or who were lost to follow-up were considered as censored. The follow-up period was defined as the time from surgery to relapse or death (for complete observations) or to the last observation (for censored cases). The Kaplan–Meier method

was used to construct DFS curves, and the log-rank test was used to test the differences in survival by covariates. Prognostic models for DFS events were constructed using univariate and multivariate Cox analyses. Median follow-up was estimated using the reverse Kalplan–Meier method [19].

We constructed a linear regression to examine the correlation between the expression of core cleavage and polyadenylation (C/P) factors and SUI for each transcript. In this model, the SUI was employed as a response variable, whereas the expression levels of core C/P factors were considered as predictors. We conducted model selection based on the Akaike Information Criterion (AIC), for which the maximal model is

$$\mathrm{Y}_i = \beta_0 + \beta_1 \mathrm{X1}_i + \beta_2 \mathrm{X2}_i + \cdots + \beta_n \mathrm{X}n_i + \varepsilon_i$$

Where i is the index for a given transcript; Y is the SUI of given transcript; and Xn represents the expression of individual C/P factor. We applied a Benjamini & Hochberg adjustment (false discovery rate, FDR) cutoff of 0.05 to linear model p-values to identify statistically significant models.

Repeated measures analysis of variance (RMANOVA) with Dunnett's t test was used to compare the cell proliferation between groups. Other Continuous variables were analyzed using the Mann-Whitney test or analysis of variance (ANOVA).

SUPPLEMENTARY FIGURE

Figure S1

Number of selected tandem 3'UTRs (based on coefficient of variation, CV) on clustering results.

3'UTR: 3' untranslated regions.

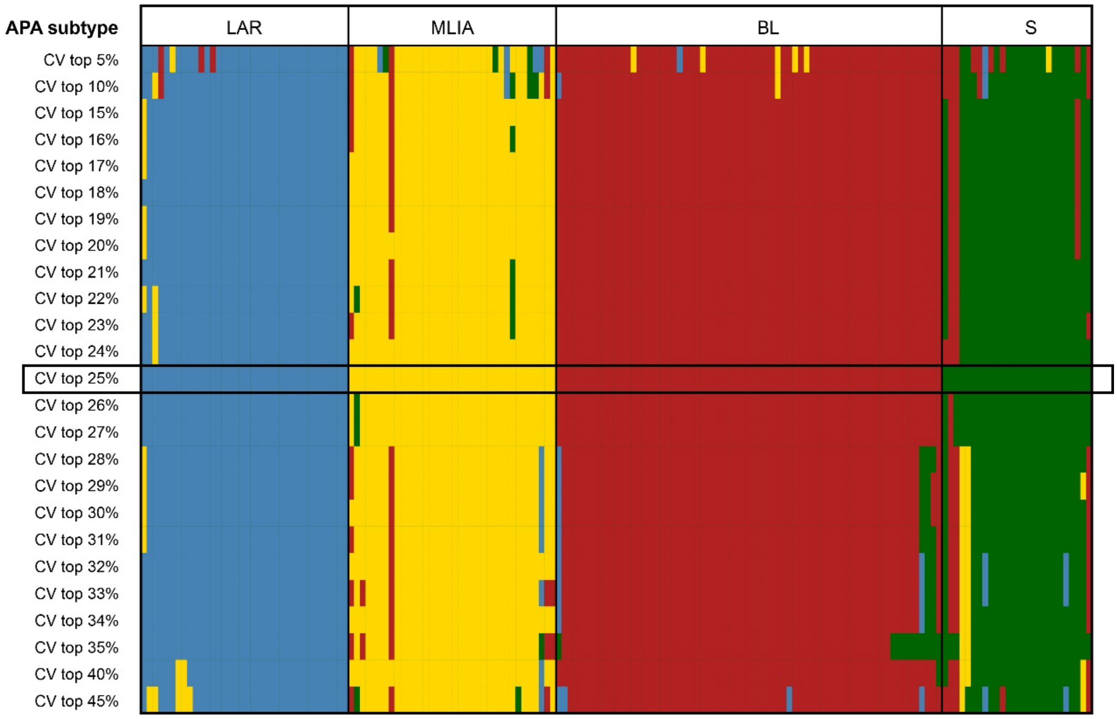

Figure S2

Bayesian change point analysis of (**A**) *FOXA1*, (**B**) *EIF4A2*, (**C**) *ERBB2* and (**D**) *TEKT4*.

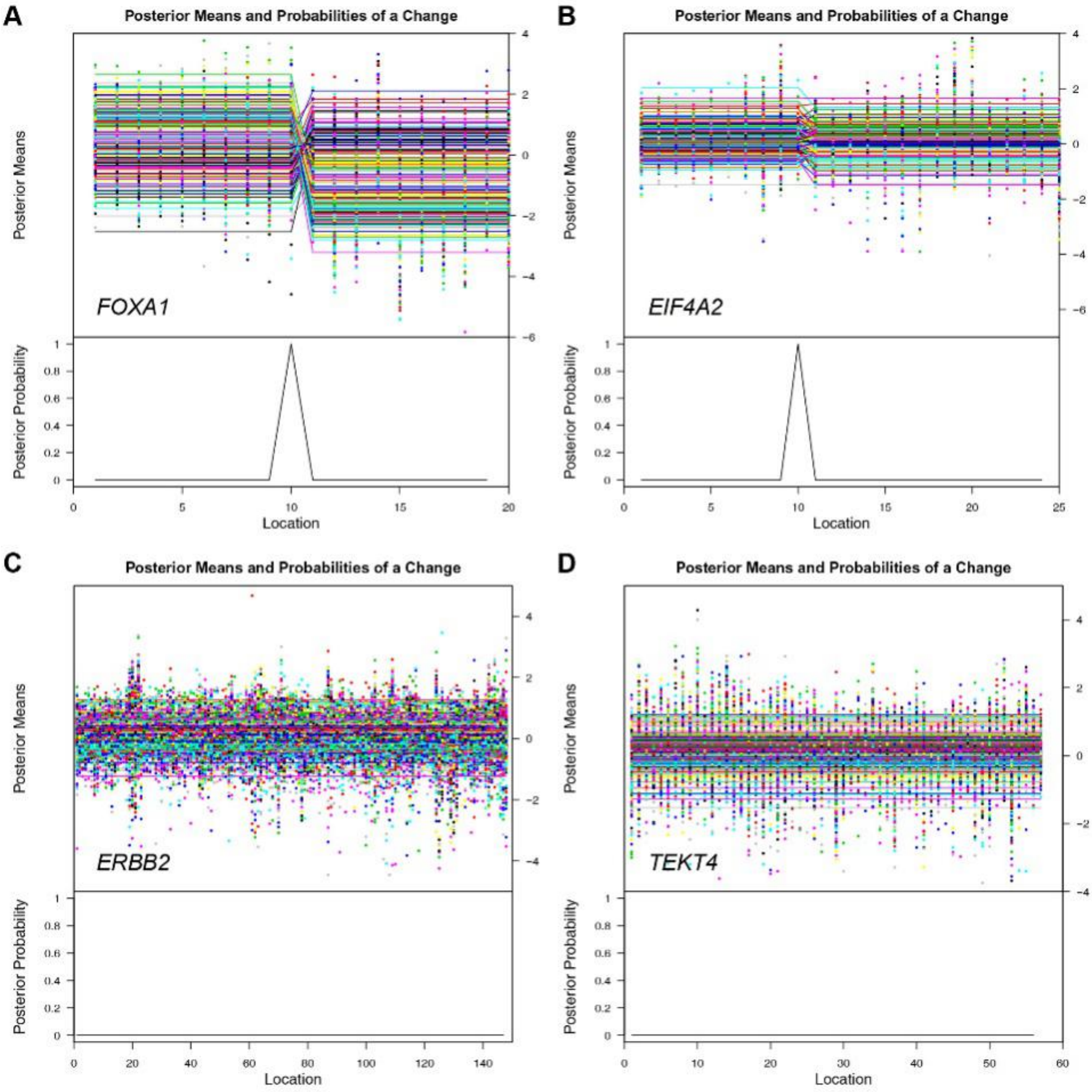

Figure S3

Flow chart of tandem 3′UTR generation.

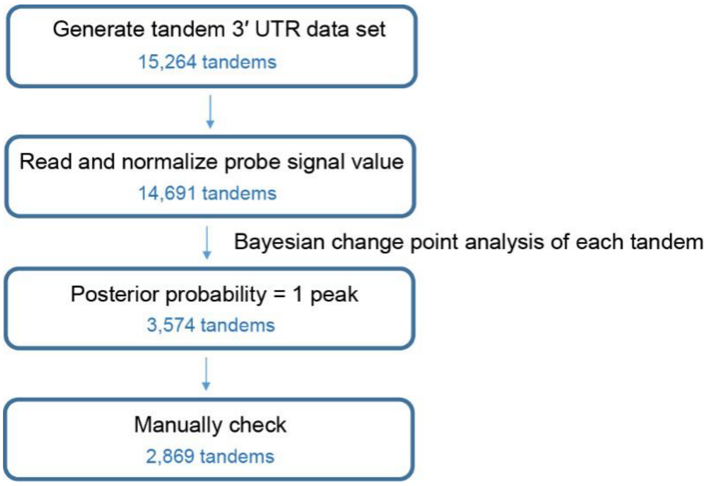

Figure S4

Alternative polyadenylation (APA) events in triple-negative breast cancer (TNBC). (A) APA events in 165 TNBCs compared with 33 normal adjacent breast tissues. Out of 1631 significant APA events, 68.5% (1118 of 1631) were 3′UTR shortening, whereas 31.5% (513 of 1631) were 3′UTR lengthening events. (**B**) Gene Ontology (GO) enrichment analysis for biological process of genes with APA events. Heatmap of enriched terms colored by *p*-value. (**C**) GO enrichment analysis for biological process of genes with APA events. Heatmap of enriched terms colored by *p*-value.

APA: alternative polyadenylation; GO: Gene Ontology.

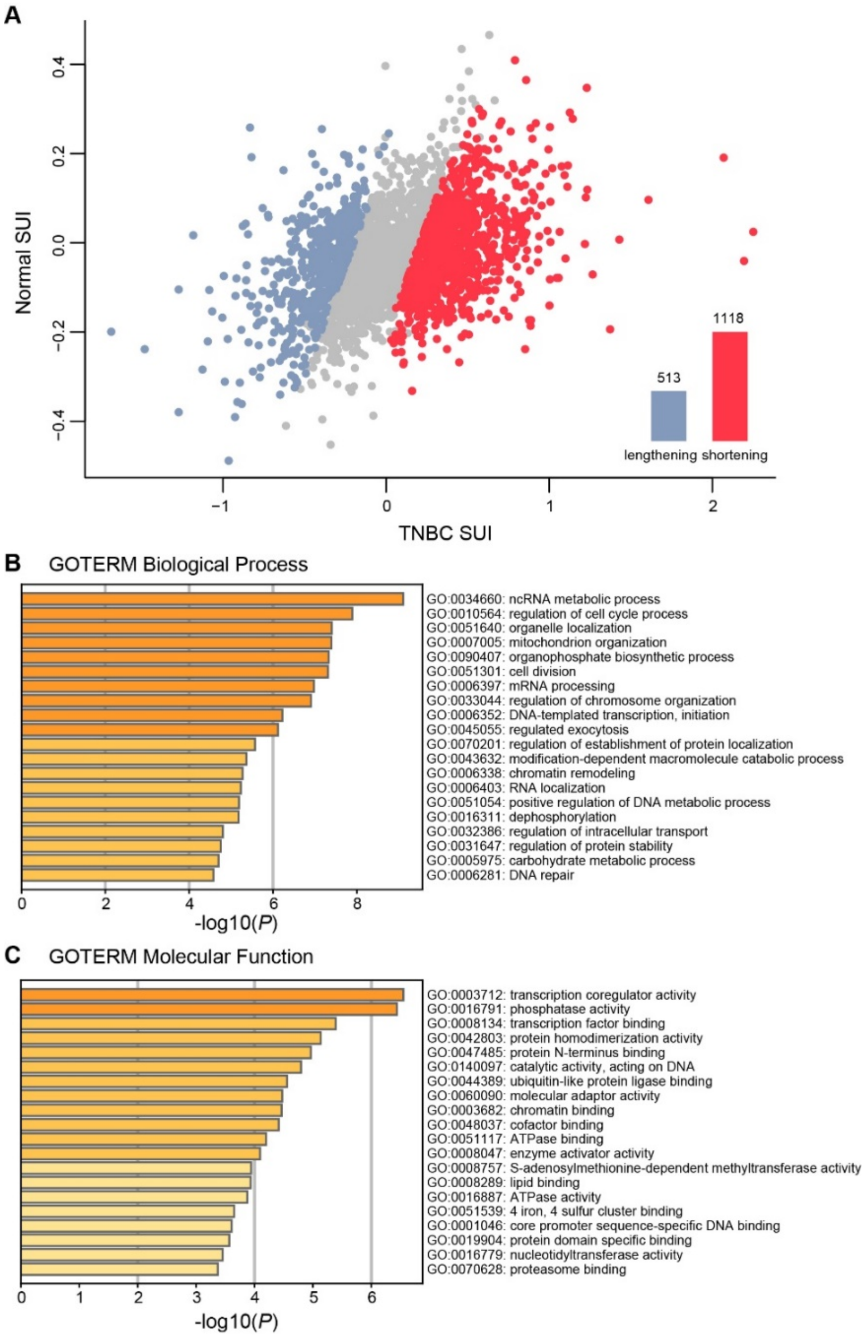

Figure S5

Spearman's correlation scatterplot of the short 3′UTR index (SUI) and the percentage distal poly(A) site usage index (PDUI) in MDA-MB-231.

PDUI: percentage distal poly(A) site usage index; SUI: short 3′UTR index.

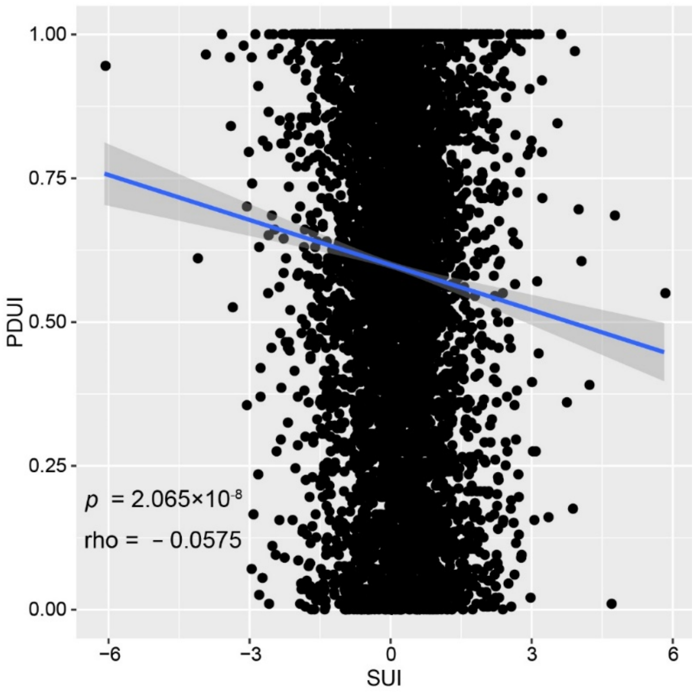

Figure S6

Distribution of the Spearman's rank correlation coefficient between percentage distal poly(A) site usage index (PDUI) and protein level across the TCGA breast cancer subjects.

PDUI: percentage distal poly(A) site usage index; TCGA: the Cancer Genome Atlas.

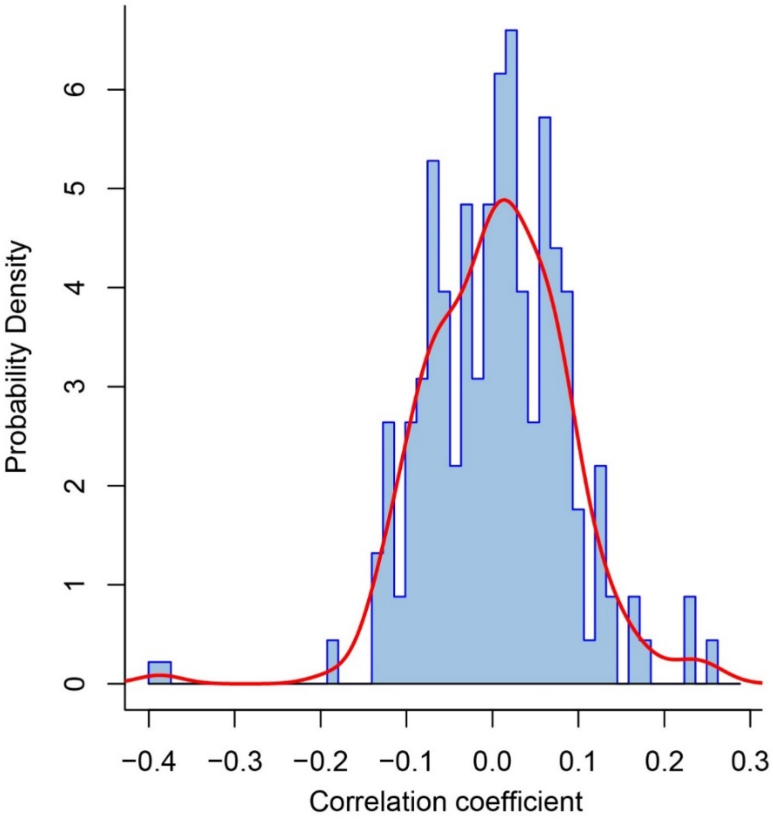

Figure S7

Differential gene expression across alternative polyadenylation (APA) subtypes of triple-negative breast cancer (TNBC). Heatmaps show relative gene expression (log2, -6 to 6) associated with proliferation, DNA damage response, myoepithelial genes, immune signal transduction, TGFβ signaling, growth factor receptors, epithelial-mesenchymal transition (EMT), Wnt signaling, stem-like, claudin-low (CL), angiogenesis, AR-driven genes, homeotic complex (HOX) genes, and cytokeratin genes across TNBC APA subtypes.

APA: alternative polyadenylation; AR: androgen receptor; BL: basal-like; EMT: epithelial-mesenchymal transition; LAR: luminal androgen receptor; MLIA: mesenchymal-like immuno-activated; S, suppressed; TNBC: triple-negative breast cancer.

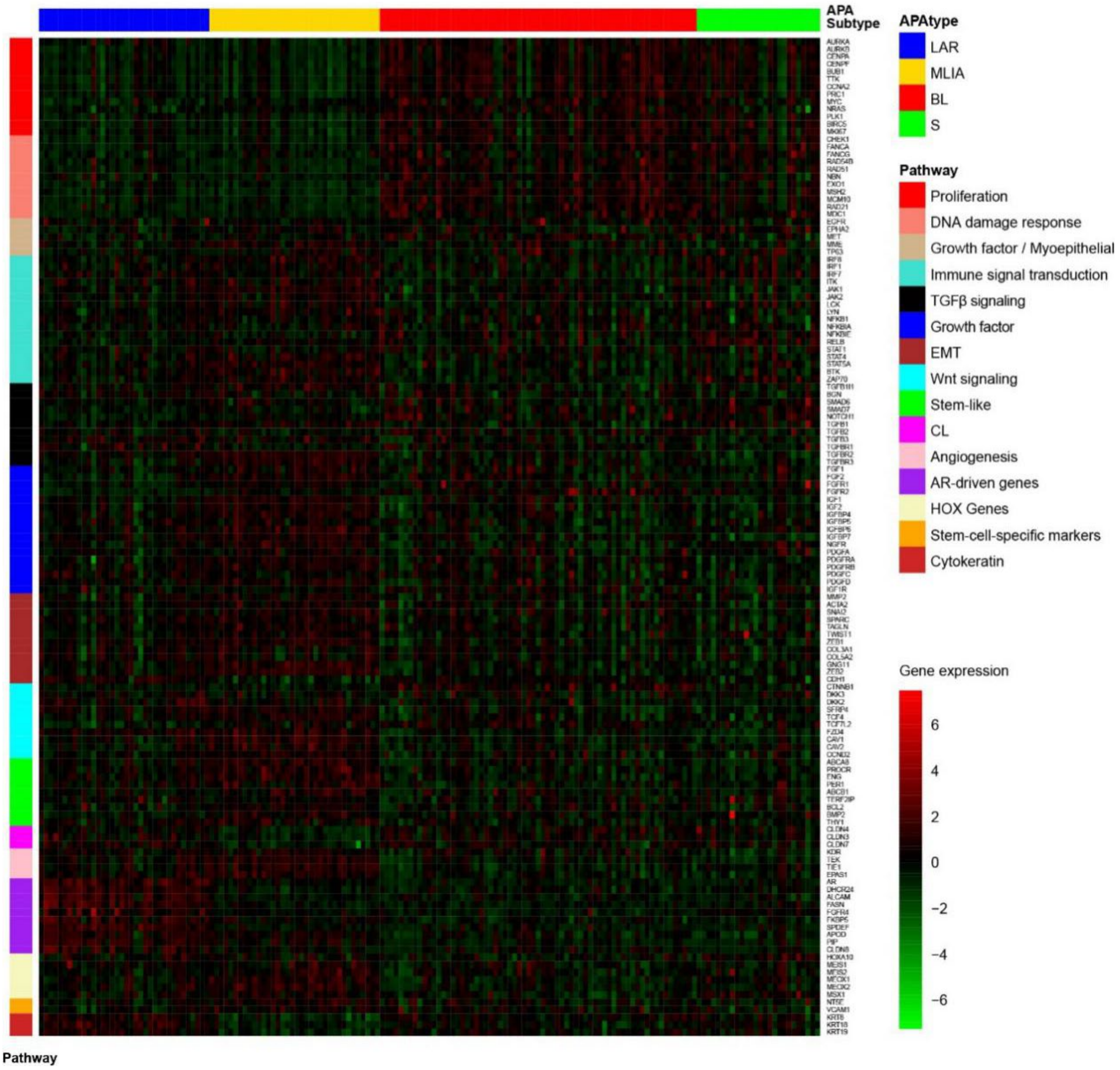

Figure S8

Differences in median short 3′UTR index (SUI) between groups were calculated using least significant difference (LSD) multiple comparisons test. ** $p < 0.01$; *** $p < 0.001$

BL: basal-like; LAR: luminal androgen receptor; LSD: least significant difference; MLIA: mesenchymal-like immune-activated; S: suppressed; SUI: short 3′UTR index.

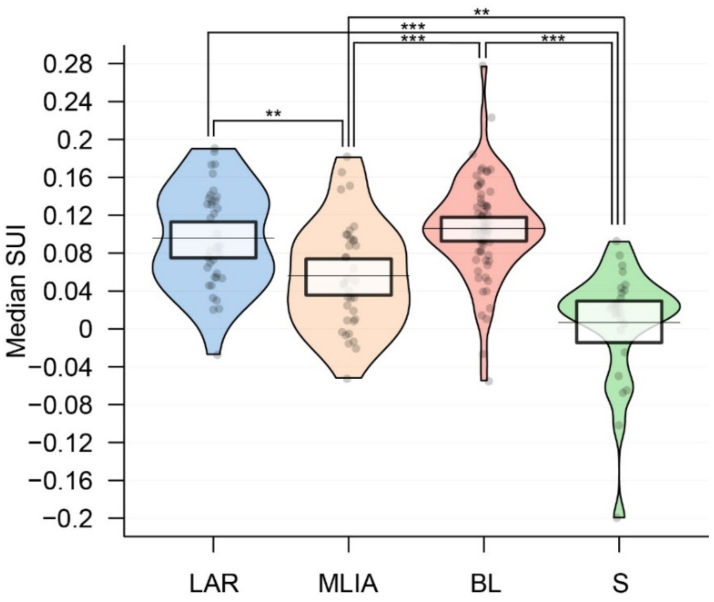

Figure S9

The enrichment analysis of genes with shortening tandem 3'UTRs in the Suppressed (S) subtype. (**A**) Heatmap of enriched terms colored by *p*-value. (**B**) Network of enriched terms colored by *p*-value, where terms containing more genes tend to have a more significant *p*-value. (**C**) Protein-protein interaction (PPI) network and molecular complex detection (MCODE) components identified in the gene list.

3'UTR: 3' untranslated region; MCODE: molecular complex detection; PPI: protein-protein interaction; S: suppressed.

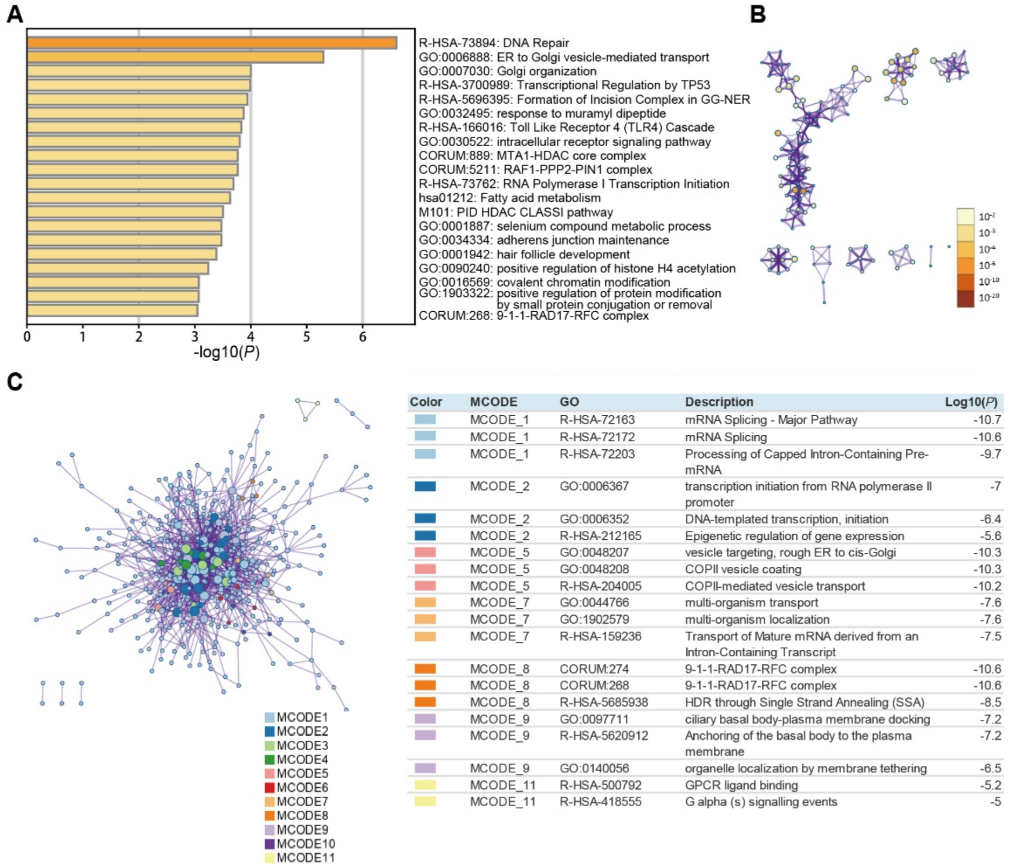

Color	MCODE	GO	Description	Log10(*P*)
	MCODE_1	R-HSA-72163	mRNA Splicing - Major Pathway	-10.7
	MCODE_1	R-HSA-72172	mRNA Splicing	-10.6
	MCODE_1	R-HSA-72203	Processing of Capped Intron-Containing Pre-mRNA	-9.7
	MCODE_2	GO:0006367	transcription initiation from RNA polymerase II promoter	-7
	MCODE_2	GO:0006352	DNA-templated transcription, initiation	-6.4
	MCODE_2	R-HSA-212165	Epigenetic regulation of gene expression	-5.6
	MCODE_5	GO:0048207	vesicle targeting, rough ER to cis-Golgi	-10.3
	MCODE_5	GO:0048208	COPII vesicle coating	-10.3
	MCODE_5	R-HSA-204005	COPII-mediated vesicle transport	-10.2
	MCODE_7	GO:0044766	multi-organism transport	-7.6
	MCODE_7	GO:1902579	multi-organism localization	-7.6
	MCODE_7	R-HSA-159236	Transport of Mature mRNA derived from an Intron-Containing Transcript	-7.5
	MCODE_8	CORUM:274	9-1-1-RAD17-RFC complex	-10.6
	MCODE_8	CORUM:268	9-1-1-RAD17-RFC complex	-10.6
	MCODE_8	R-HSA-5685938	HDR through Single Strand Annealing (SSA)	-8.5
	MCODE_9	GO:0097711	ciliary basal body-plasma membrane docking	-7.2
	MCODE_9	R-HSA-5620912	Anchoring of the basal body to the plasma membrane	-7.2
	MCODE_9	GO:0140056	organelle localization by membrane tethering	-6.5
	MCODE_11	R-HSA-500792	GPCR ligand binding	-5.2
	MCODE_11	R-HSA-418555	G alpha (s) signalling events	-5

Figure S10

The enrichment analysis of genes with lengthening tandem 3'UTRs in the S subtype. (**A**) Heatmap of enriched terms colored by *p*-value. (**B**) Network of enriched terms colored by *p*-value, where terms containing more genes tend to have a more significant *p*-value. (**C**) Protein-protein interaction (PPI) network and MCODE components identified in the gene list.

3'UTR: 3' untranslated region; MCODE: molecular complex detection; PPI: protein-protein interaction.

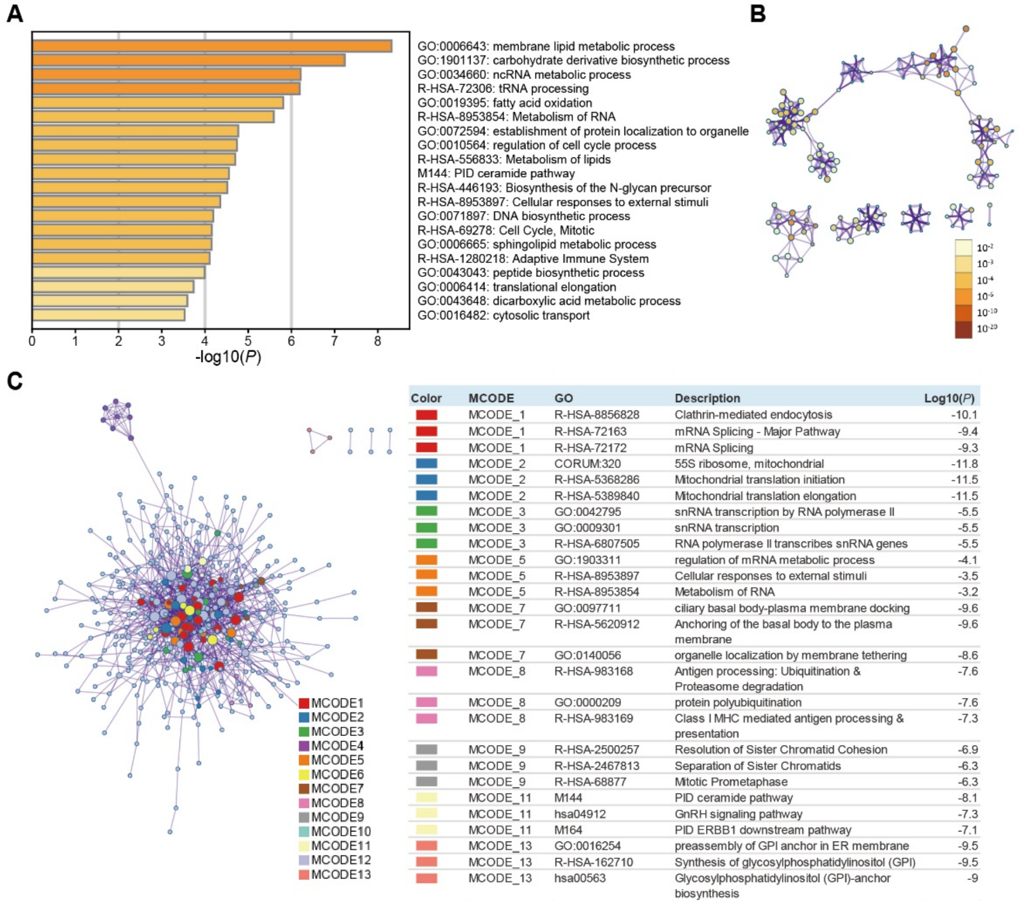

Color	MCODE	GO	Description	Log10(*P*)
	MCODE_1	R-HSA-8856828	Clathrin-mediated endocytosis	-10.1
	MCODE_1	R-HSA-72163	mRNA Splicing - Major Pathway	-9.4
	MCODE_1	R-HSA-72172	mRNA Splicing	-9.3
	MCODE_2	CORUM:320	55S ribosome, mitochondrial	-11.8
	MCODE_2	R-HSA-5368286	Mitochondrial translation initiation	-11.5
	MCODE_2	R-HSA-5389840	Mitochondrial translation elongation	-11.5
	MCODE_3	GO:0042795	snRNA transcription by RNA polymerase II	-5.5
	MCODE_3	GO:0009301	snRNA transcription	-5.5
	MCODE_3	R-HSA-6807505	RNA polymerase II transcribes snRNA genes	-5.5
	MCODE_5	GO:1903311	regulation of mRNA metabolic process	-4.1
	MCODE_5	R-HSA-8953897	Cellular responses to external stimuli	-3.5
	MCODE_5	R-HSA-8953854	Metabolism of RNA	-3.2
	MCODE_7	GO:0097711	ciliary basal body-plasma membrane docking	-9.6
	MCODE_7	R-HSA-5620912	Anchoring of the basal body to the plasma membrane	-9.6
	MCODE_7	GO:0140056	organelle localization by membrane tethering	-8.6
	MCODE_8	R-HSA-983168	Antigen processing: Ubiquitination & Proteasome degradation	-7.6
	MCODE_8	GO:0000209	protein polyubiquitination	-7.6
	MCODE_8	R-HSA-983169	Class I MHC mediated antigen processing & presentation	-7.3
	MCODE_9	R-HSA-2500257	Resolution of Sister Chromatid Cohesion	-6.9
	MCODE_9	R-HSA-2467813	Separation of Sister Chromatids	-6.3
	MCODE_9	R-HSA-68877	Mitotic Prometaphase	-6.3
	MCODE_11	M144	PID ceramide pathway	-8.1
	MCODE_11	hsa04912	GnRH signaling pathway	-7.3
	MCODE_11	M164	PID ERBB1 downstream pathway	-7.1
	MCODE_13	GO:0016254	preassembly of GPI anchor in ER membrane	-9.5
	MCODE_13	R-HSA-162710	Synthesis of glycosylphosphatidylinositol (GPI)	-9.5
	MCODE_13	hsa00563	Glycosylphosphatidylinositol (GPI)-anchor biosynthesis	-9

Figure S11

Subtype-specific alternative polyadenylation (APA) and analysis of their co-expressed mRNAs. The subtype with the highest short 3′UTR index (SUI) was computed using the R package 'limma'. (**A**) Luminal androgen receptor (LAR); (**B**) mesenchymal-like immune-activated (MLIA); (**C**) basal-like (BL); (**D**) suppressed (S). Yellow circle, tandem 3′UTR; red circle, up-regulated mRNA; blue circle, down-regulated mRNA. *** $p < 0.001$, ** $p < 0.01$.

APA: alternative polyadenylation; BL: basal-like; LAR: luminal androgen receptor; MLIA: mesenchymal-like immune-activated; S: suppressed; SUI: short 3′UTR index; TNBC: triple-negative breast cancer.

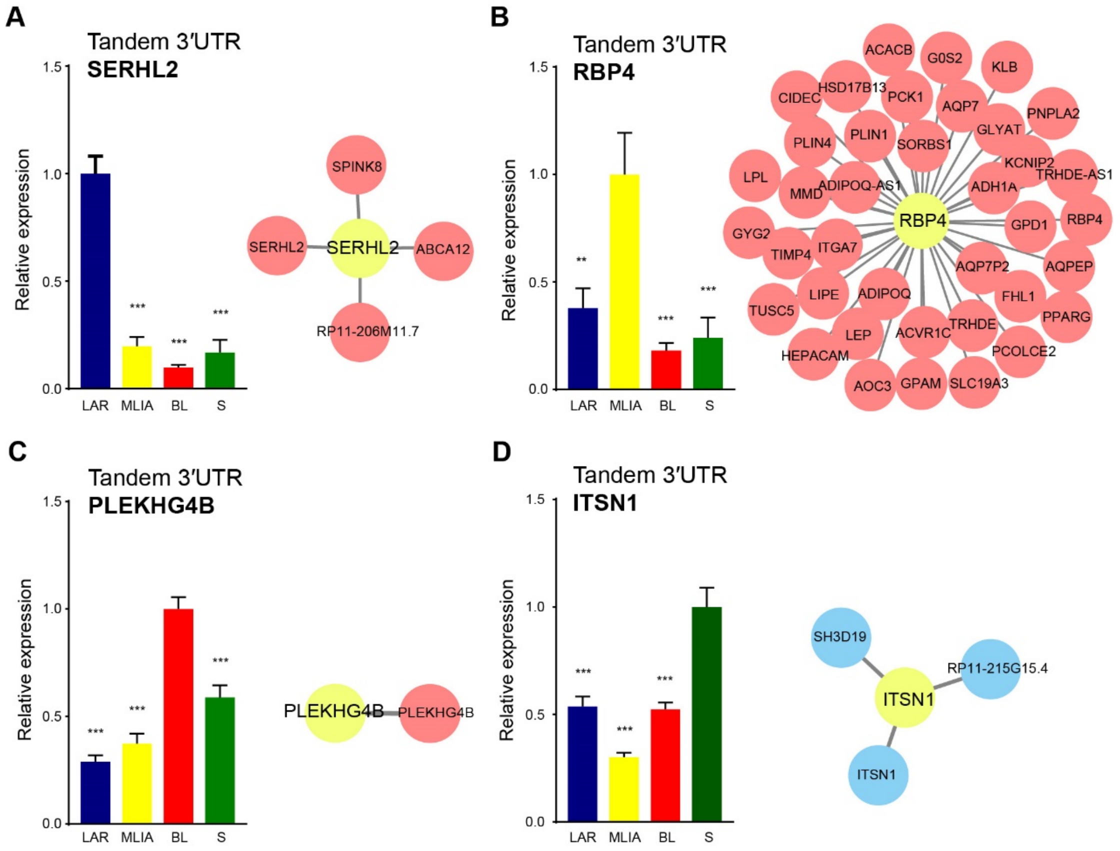

Figure S12

Sankey diagram reveals the relationship between Lehmann subtypes and APA subtypes.

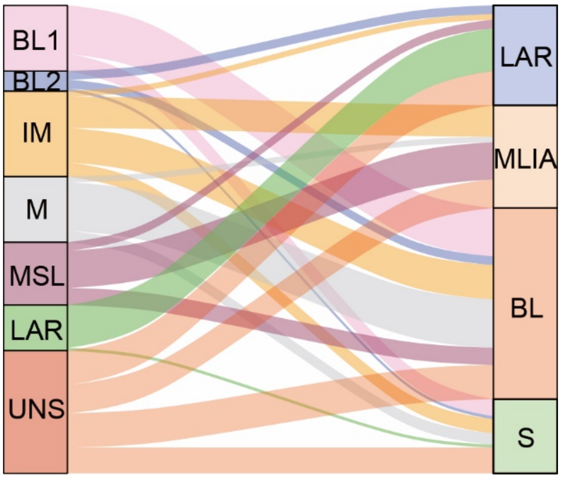

Figure S13

Triple-negative breast cancer subtypes differentially stain for Ki-67 by immunohistochemistry (Tukey's multiple comparisons test). ** $p < 0.01$; *** $p < 0.001$

BL: basal-like; LAR: luminal androgen receptor; MLIA: mesenchymal-like immune-activated; S: suppressed.

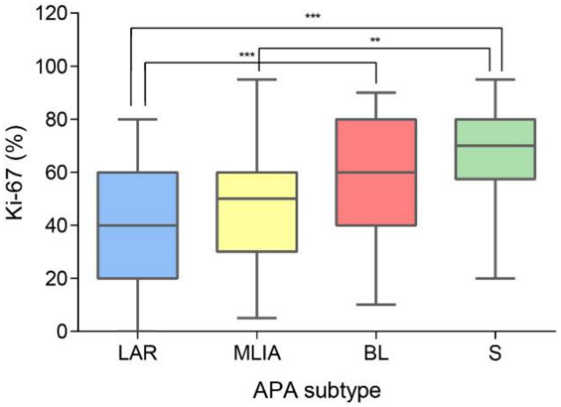

Figure S14

Increased gene expression of core 3′ cleavage and polyadenylation (C/P) factors in triple-negative breast cancer (TNBC). (**A**) Waterfall plot of the gene expression of core 3′ C/P factors in TNBC. Data are presented as the mean ± SEM (standard error of mean). (**B**) Comparison of the distribution of fold-change in core 3′ C/P factors and background gene set expression levels between TNBC and normal tissues (Kolmogorov-Smirnov test). ** $p < 0.05$; ** $p < 0.01$; *** $p < 0.001$

C/P: cleavage and polyadenylation; SEM: standard error of the mean; TNBC: triple-negative breast cancer.

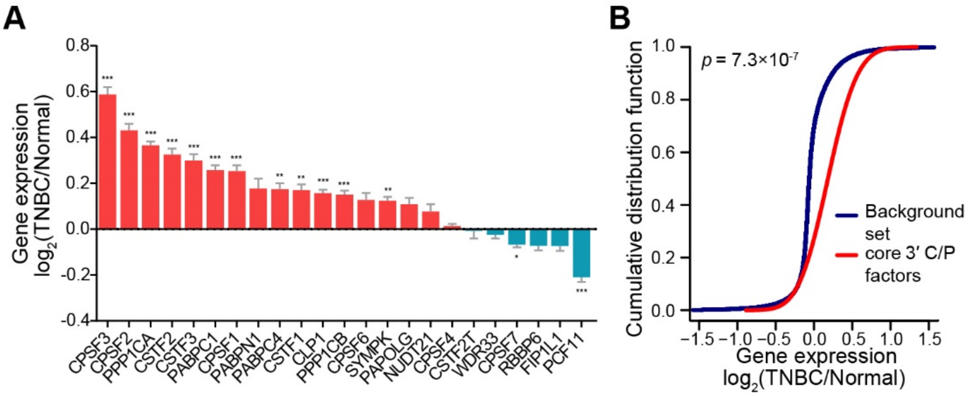

Figure S15

The heatmap of gene expression fold change of core 3′ processing factors across alternative polyadenylation (APA) subtype. Each rectangle represents the mean $\log_2$ fold change between cancer and paired normal tissue of one factor in one APA subtype. A factor is considered differentially expressed if the false-discovery rate from 'limma' [17, 18] is < 0.05 and the mean absolute fold change is > 1.2. The rainbow color map indicates the fold change of significantly upregulated and downregulated genes. White boxes represent non-significant genes.

APA: alternative polyadenylation.

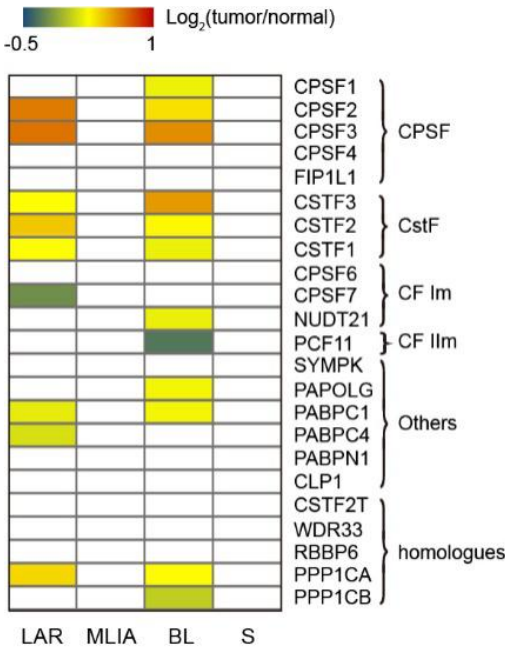

Figure S16

Number and percent of alternative polyadenylation (APA) events correlated with expression levels of core 3′ cleavage and polyadenylation factors.

APA: alternative polyadenylation.

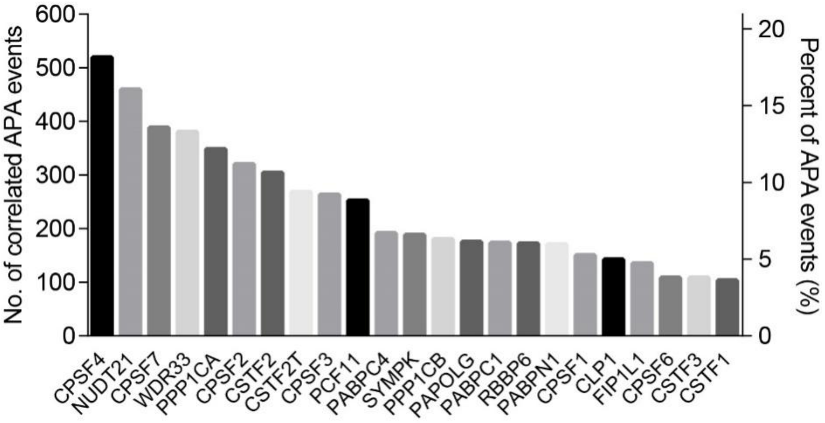

Figure S17

Correlations between alternative polyadenylation (APA) events of clinically actionable genes and selected 3′cleavage and polyadenylation factors. (**A**) Selected correlations (Pearson correlation test) between APA events of clinically actionable genes and *CPSF1* expression level (left: *PIK3C2G*; middle: *IL21A*; right: *RAD51D*). (**B**) Selected correlations (Pearson correlation test) between APA events of clinically actionable genes and *PABPN1* expression level (left: *NUDT5*; middle: *HDAC7*; right: *HDAC1*).

APA, alternative polyadenylation.

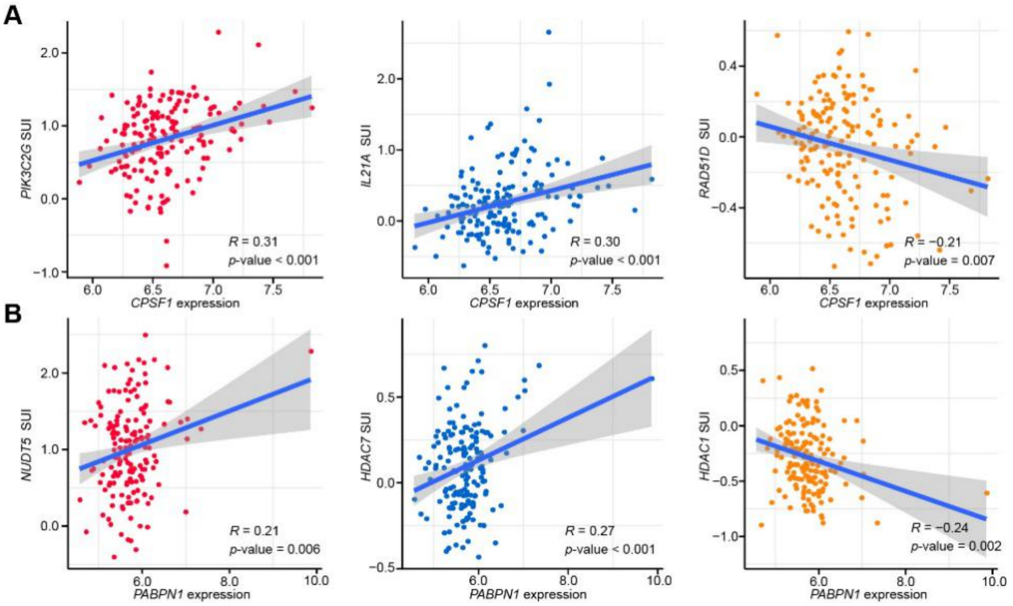

Figure S18

Short time-series analysis of shRNA library data from next-generation sequencing using the Short Time-series Expression Miner (STEM). (**A**) MDA-MB-231. (**B**) MDA-MB-468.

STEM: Short Time-series Expression Miner.

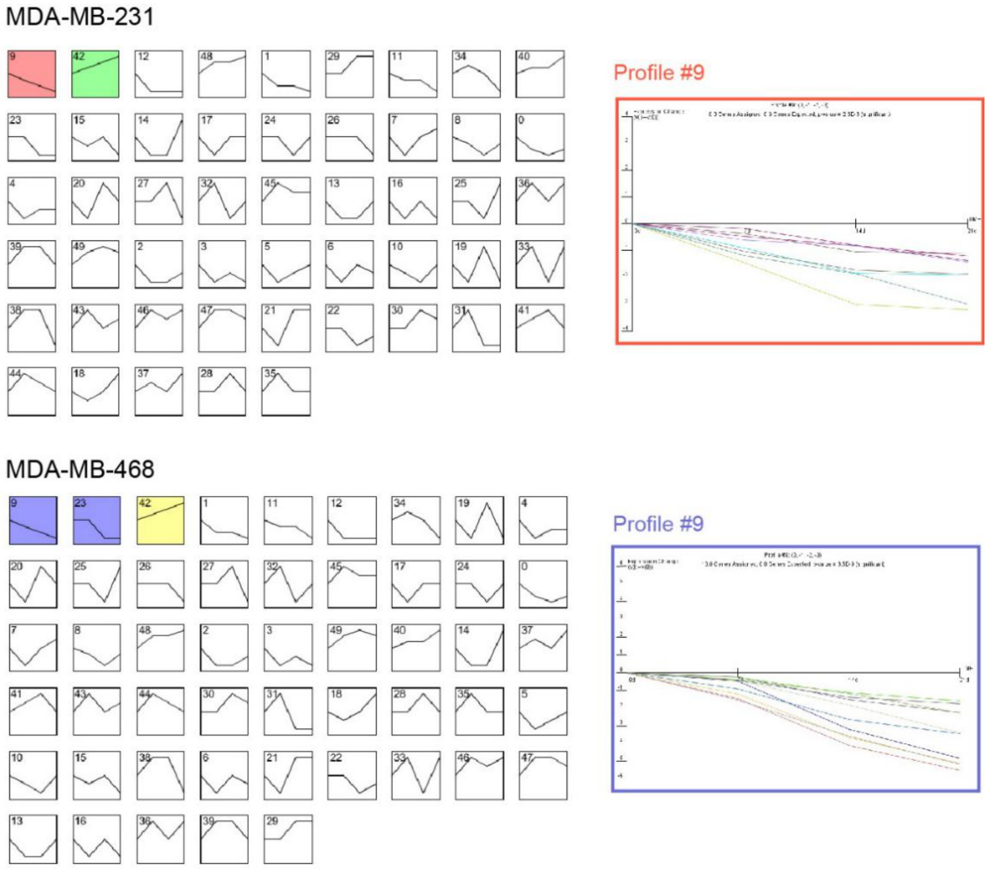

Figure S19

CPSF1 knockdown increased the (**A**) apoptosis rate and resulted in (**B**) G1/S arrest in MDA-MB-468 cells (n = 3).

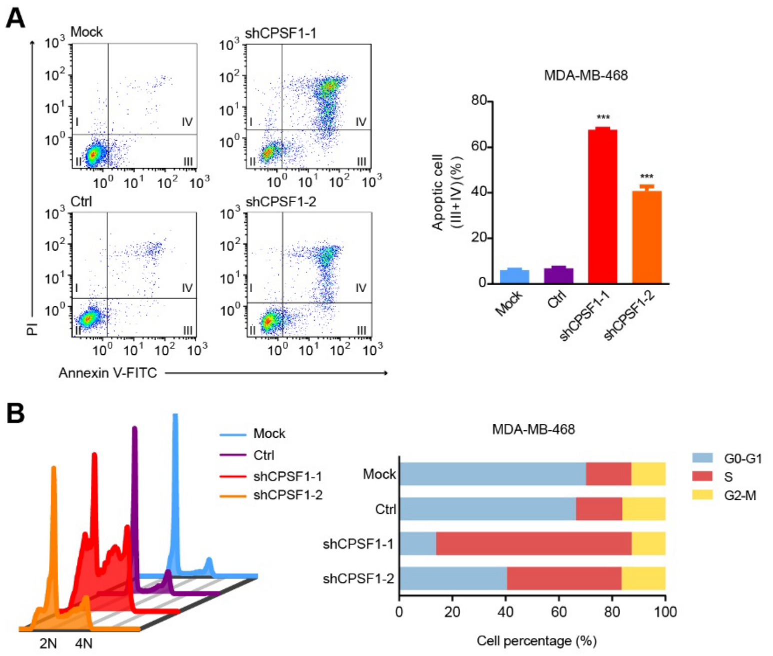

Figure S20

The clustering of differentially expressed genes in CPSF1 knockdown and control MDA-MB-231 cell lines (fold change ≥ 2, false discovery rate [FDR] < 0.05).

FDR: false discovery rate.

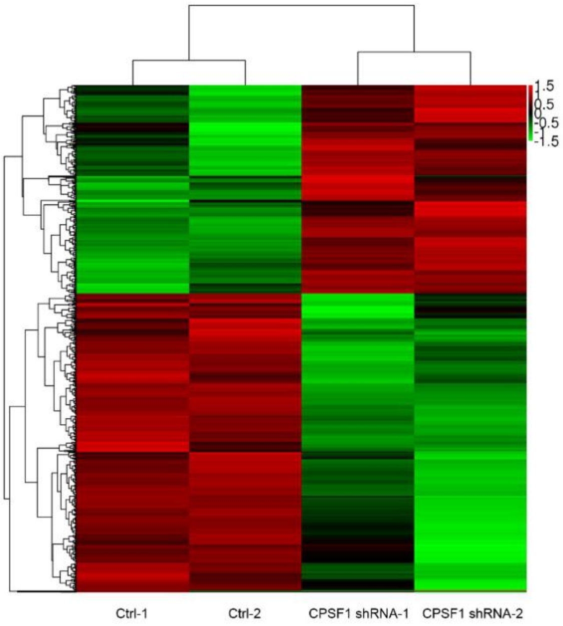

Figure S21

Venn diagram of alternative polyadenylation (APA) events. (**A**) Venn diagram of transcripts with shortened 3'UTR in TNBC samples and transcripts with lengthened 3'UTR in CPSF1-depleted TNBC cells. (**B**) Venn diagram of transcripts with lengthened 3'UTR in TNBC samples and transcripts with shortened 3'UTR in CPSF1-depleted TNBC cells. (**C**) Venn diagram of transcripts with shortened 3'UTR in TNBC samples and transcripts with lengthened 3'UTR in PABPN1-depleted TNBC cells. (**D**) Venn diagram of transcripts with lengthened 3'UTR in TNBC samples and transcripts with shortened 3'UTR in PABPN1-depleted TNBC cells. The boxes represent the intersection sets of genes with APA events.

3'UTR: 3' untranslated region; TNBC: triple-negative breast cancer.

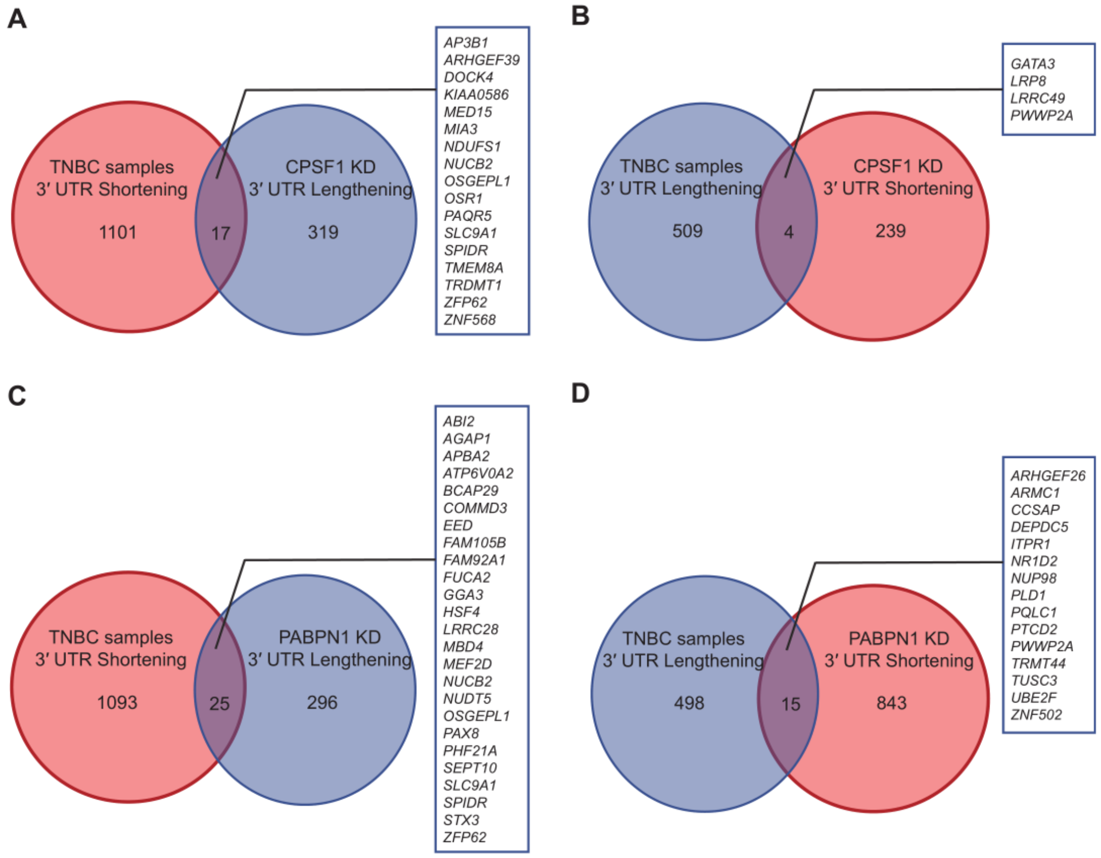

Figure S22

Kaplan–Meier survival analysis using the Cancer Genome Atlas (TCGA) cohort. (**A**) SPIDR; (**B**) MTA3; (**C**) MIA3.

TCGA: the Cancer Genome Atlas.

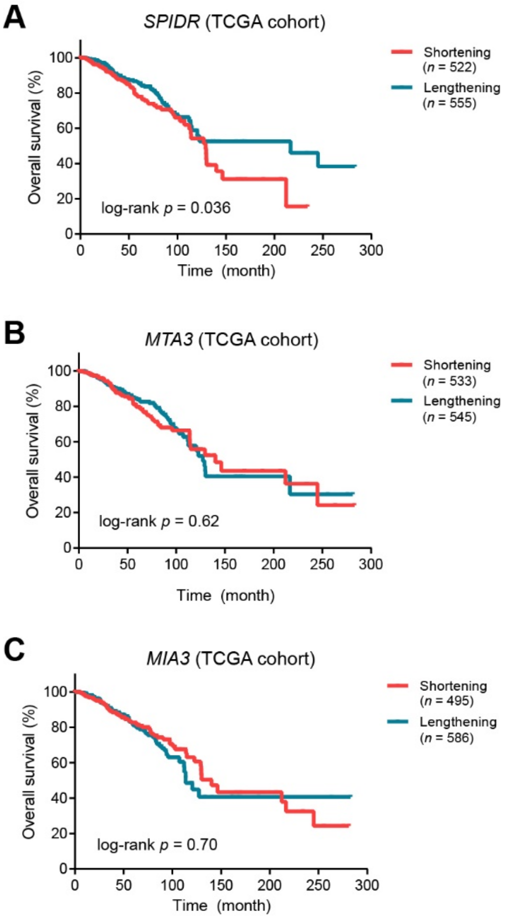

Figure S23

PABPN1 depletion results in decreased proliferation. (**A**) Western blot analysis of MDA-MB-231 and MDA-MB-468 lysates infected with control and shRNA lentivirus targeting PABPN1. (**B**) Growth of MDA-MB-231 cells and MDA-MB-468 cells was measured after infection with PABPN1 shRNA lentivirus compared with control shRNA lentivirus. The results shown are the mean ± standard deviation (s.d.) ($n = 3$). *** $p < 0.001$.

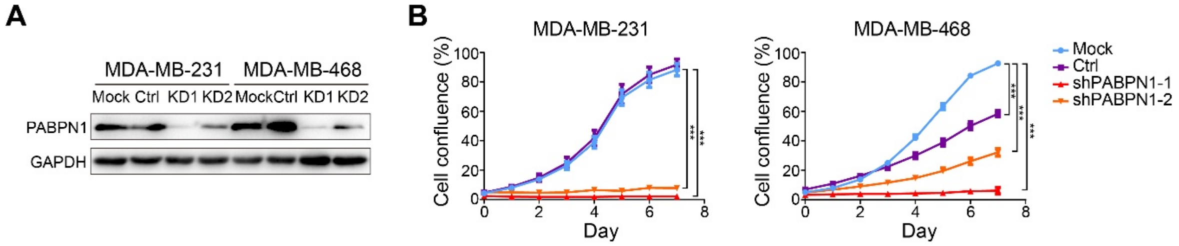

Figure S24

PABPN1 depletion results in enhanced apoptosis in triple-negative breast cancer cell lines. (**A**) MDA-MB-231. (**B**) MDA-MB-468.

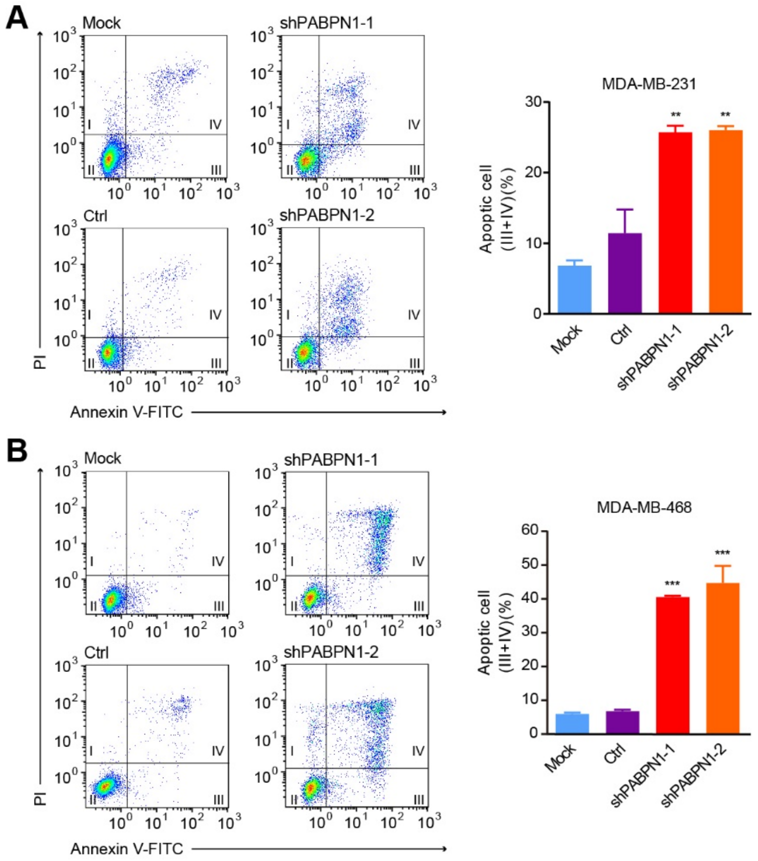

Figure S25

Cell cycle analysis of PABPN1-depleted triple-negative breast cancer cell lines. (**A**) MDA-MB-231. (**B**) MDA-MB-468.

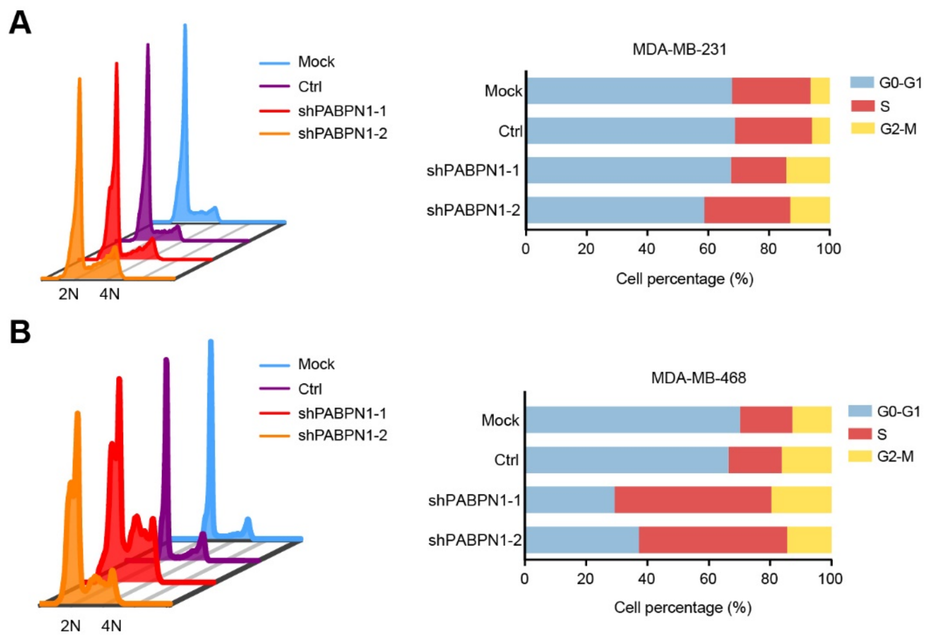

Figure S26

The clustering of differential expressed genes in PABPN1 knockdown and control MDA-MB-231 cell lines (fold change ≥ 2, false discovery rate [FDR] < 0.05).

FDR: false discovery rate.

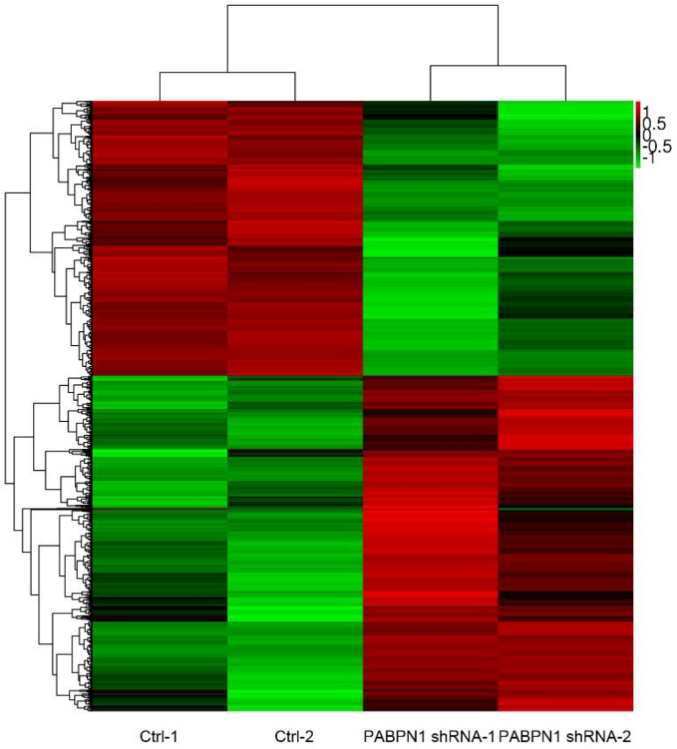

.

Figure S27

Enriched pathways in PABPN1 knockdown MDA-MB-231.

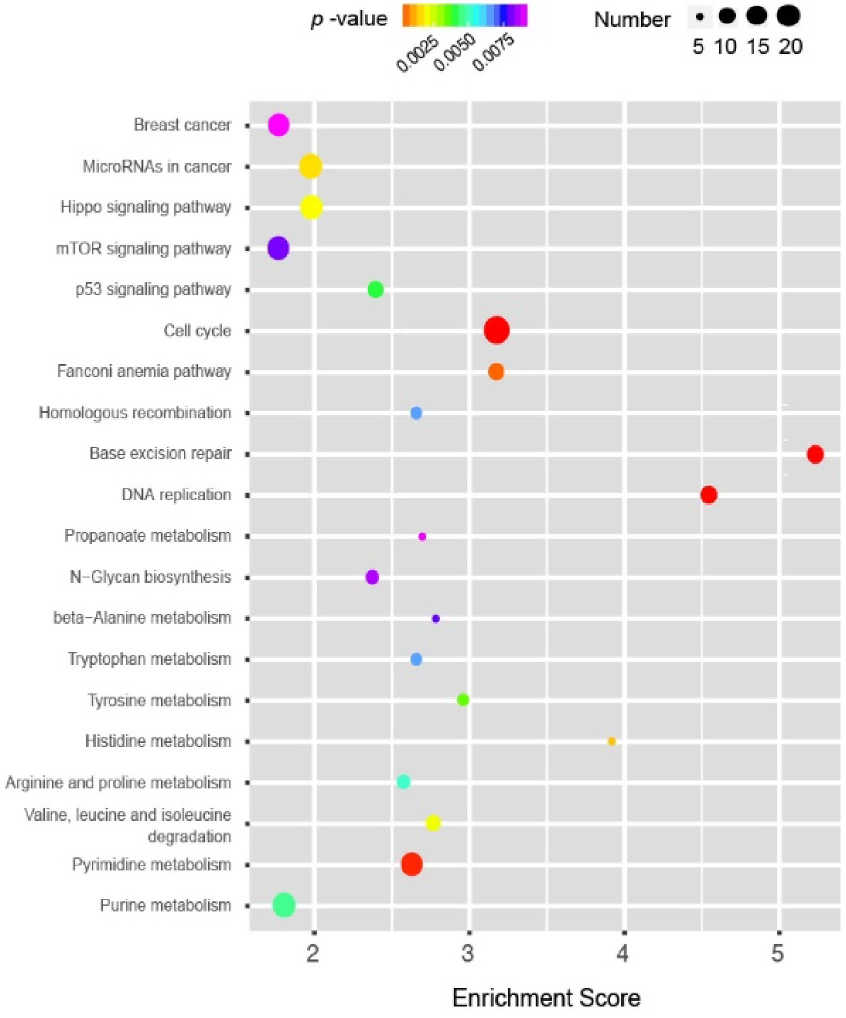

Figure S28

Alternative polyadenylation (APA) events in PABPN1 knockdown MDA-MB-231. (**A**) Scatterplot of the percentage of distal poly(A) sites usage index (PDUI) in the control and PABPN1 knockdown groups where mRNAs were significantly shortened (n = 858) and lengthened (n = 321) after PABPN1 knockdown. (**B**) Correlation between distal poly(A) site usage and gene expression levels of control and PABPN1-knockdown MDA-MB-231. (**C**) Representative RNA-seq density plots along with ΔPDUI values for genes whose 3′UTRs were lengthened (*MMS22L*, *DHX36* and *MUM1*) and shortened (*PHF21A*) in response to PABPN1 knockdown.

APA: alternative polyadenylation.

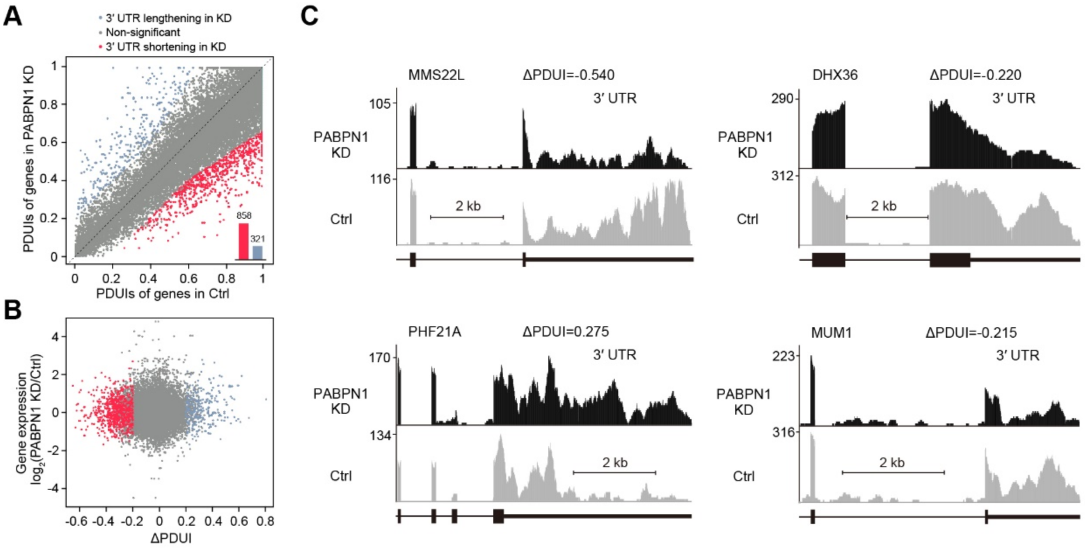

Figure S29

Kaplan–Meier survival analysis using the Cancer Genome Atlas (TCGA) cohort. (**A**) *DHX36*; (**B**) *PHF21A*; (**C**) *MUM1*.

TCGA: the Cancer Genome Atlas.

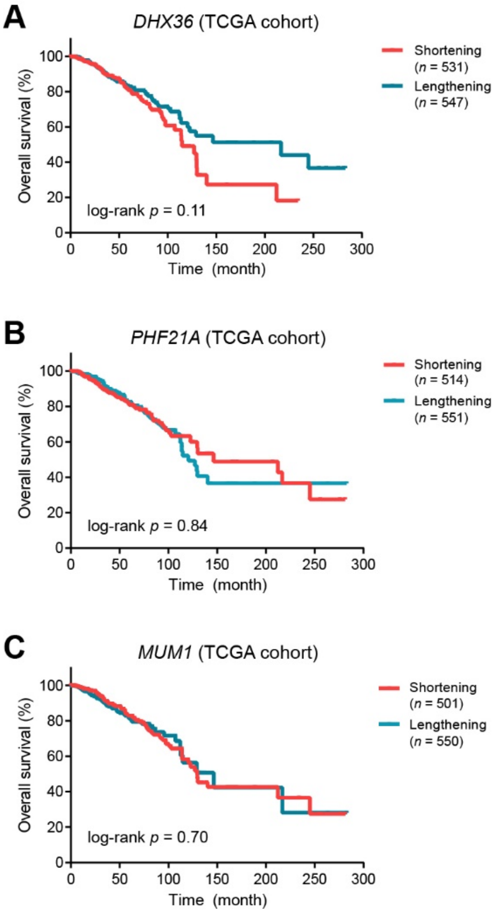

References

1. Bengtsson H, Wirapati P, Speed TP. A single-array preprocessing method for estimating full-resolution raw copy numbers from all Affymetrix genotyping arrays including GenomeWideSNP 5 & 6. Bioinformatics. 2009; 25: 2149-56.
2. Subramanian A, Tamayo P, Mootha VK, Mukherjee S, Ebert BL, Gillette MA, *et al*. Gene set enrichment analysis: a knowledge-based approach for interpreting genome-wide expression profiles. Proc Natl Acad Sci U S A. 2005; 102: 15545-50.
3. Lehmann BD, Bauer JA, Chen X, Sanders ME, Chakravarthy AB, Shyr Y, *et al*. Identification of human triple-negative breast cancer subtypes and preclinical models for selection of targeted therapies. J Clin Invest. 2011; 121: 2750-67.
4. Zhou Y, Zhou B, Pache L, Chang M, Khodabakhshi AH, Tanaseichuk O, *et al*. Metascape provides a biologist-oriented resource for the analysis of systems-level datasets. Nat Commun. 2019; 10: 1523.
5. Pujana MA, Han JD, Starita LM, Stevens KN, Tewari M, Ahn JS, *et al*. Network modeling links breast cancer susceptibility and centrosome dysfunction. Nat Genet. 2007; 39: 1338-49.
6. Shannon P, Markiel A, Ozier O, Baliga NS, Wang JT, Ramage D, *et al*. Cytoscape: a software environment for integrated models of biomolecular interaction networks. Genome Res. 2003; 13: 2498-504.
7. Smyth GK. Linear models and empirical bayes methods for assessing differential expression in microarray experiments. Stat Appl Genet Mol Biol. 2004; 3: Article3.
8. Ye FG, Song CG, Cao ZG, Xia C, Chen DN, Chen L, *et al*. Cytidine Deaminase Axis Modulated by miR-484 Differentially Regulates Cell Proliferation and Chemoresistance in Breast Cancer. Cancer Res. 2015; 75: 1504-15.
9. Kim D, Langmead B, Salzberg SL. HISAT: a fast spliced aligner with low memory requirements. Nat Methods. 2015; 12: 357-60.
10. Roberts A, Trapnell C, Donaghey J, Rinn JL, Pachter L. Improving RNA-Seq expression estimates by correcting for fragment bias. Genome Biol. 2011; 12: R22.
11. Kanehisa M. Toward understanding the origin and evolution of cellular organisms. Protein Sci. 2019; 28: 1947-51.
12. Kanehisa M, Sato Y, Furumichi M, Morishima K, Tanabe M. New approach for understanding genome variations in KEGG. Nucleic Acids Res. 2019; 47: D590-D5.
13. Kanehisa M, Goto S. KEGG: kyoto encyclopedia of genes and genomes. Nucleic Acids Res. 2000; 28: 27-30.
14. Xia Z, Donehower LA, Cooper TA, Neilson JR, Wheeler DA, Wagner EJ, *et al*. Dynamic analyses of alternative polyadenylation from RNA-seq reveal a 3'-UTR landscape across seven tumour types. Nat Commun. 2014; 5: 5274.
15. Masamha CP, Xia Z, Yang J, Albrecht TR, Li M, Shyu AB, *et al*. CFIm25 links alternative polyadenylation to glioblastoma tumour suppression. Nature. 2014; 510: 412-6.
16. Feng X, Li L, Wagner EJ, Li W. TC3A: The Cancer 3' UTR Atlas. Nucleic Acids Res. 2018; 46: D1027-D30.
17. Phipson B, Lee S, Majewski IJ, Alexander WS, Smyth GK. Robust Hyperparameter Estimation Protects against Hypervariable Genes And Improves Power To Detect Differential Expression. The annals of applied statistics. 2016; 10: 946-63.
18. Ritchie ME, Phipson B, Wu D, Hu Y, Law CW, Shi W, *et al*. limma powers differential expression

analyses for RNA-sequencing and microarray studies. Nucleic Acids Res. 2015; 43: e47.
19. Schemper M, Smith TL. A note on quantifying follow-up in studies of failure time. Control Clin Trials. 1996; 17: 343-6.
